# Build Your Own Eye: A Method for Teaching Ocular Anatomy and Pathophysiology

**DOI:** 10.21980/J8GS8W

**Published:** 2020-07-15

**Authors:** Alaina Brinley Rajagopal, Mark Joseph Slader, Megan Boysen Osborn

**Affiliations:** * University of California, Irvine, Department of Emergency Medicine, Orange, CA

## Abstract

**Audience:**

Residents and medical students

**Introduction:**

The eye is a critical, but often neglected, part of medical learning. This team-based learning (TBL) module was developed for emergency medicine residents and medical students; however, is applicable to any learner who should know basic eye anatomy and pathology. Emergency medicine teams, primary care providers, and ophthalmologists are most likely to encounter ocular emergencies.^1^–^3^ These emergencies are uncommon but quite dire when they occur and can result in permanent disability and life-changing morbidity.^2^, ^4^ It is critical that medical practitioners who are exposed to these types of emergencies are well prepared to evaluate and treat them.

To fully understand how pathology affects the eye, it is critical that learners understand the anatomy and physiology of the eye.^5^, ^6^ Many diagnoses are associated with specific parts of ocular anatomy;^5^, ^6^ therefore, teaching pathology in an anatomy-based lesson will help learners understand the physiology. This lesson teaches learners about physiology and pathology in a systemic, anatomically oriented way.

**Educational Objectives:**

By the end of this session the participant will be able to:

1. Describe basic anatomy of the eye.
2. Build a basic model of the eye.
3. Identify which diseases are associated with which parts of the eye.
4. Identify the pathophysiology behind diseases of the eye.
5. Name correct treatment for each disease.

**Educational Methods:**

The “build your own eye” lesson was taught as a classic team-based learning (cTBL) exercise. The modality of TBL with hands-on construction of an eye allow for social learning, competition and spatial learning related to anatomy. The creation of an eye allows residents to fully understand ocular anatomy which is not as evident when a two-dimensional paper image is used. Some learners need tactical stimuli for better understanding. This aspect of the exercise was focused on using alternative modalities to enhance spatial learning. These concepts are reinforced by the GRAT and IRAT portions of the exercise which tend to multiple choice learners. The fill-in the-blank aspect of the exercise requires recall and research to match the three-dimensional eye parts with pathology.

**Research Methods:**

Learners were given the opportunity to complete an anonymous survey. Verbal feedback was also obtained from learners during the lesson. The survey asked learners questions about the effectiveness and value of the exercise, whether the content was applicable to work in the emergency department, whether this exercise should be kept as a part of the curriculum, and whether there was any practice-changing information. Learners enjoyed the competitive aspects of the exercise and also noted that they felt much more comfortable with ocular anatomy and pathology after the lesson.

**Results:**

Learners felt that the ocular team-based learning module was effective in teaching more about the eye in an atypical way. Some learners felt that an explanation in advance of the eye building aspects of the project may have been helpful so they could have brought supplies from home. Other learners felt that they would not have brought supplies from home; thus no explanation was necessary.

**Discussion:**

Learners seemed to enjoy the experience. The competitive aspects of the TBL, where the eye models were judged for accuracy, creativity, and appearance as well as the correct answers on the worksheet, seemed to enhance learner enjoyment and engagement. Learners felt that enough time was provided for the exercise. While some learners would have preferred an explanation in advance of the project in order to bring supplies from home, others felt that this was not necessary. Educators should determine what would be preferred by their particular learning group for future implementations.

**Topics:**

Eye lid, tear duct, cornea, conjunctiva, pupil, iris, lens, anterior chamber, vitreous body, posterior chamber, retina, macula, choroid, optic disc, optic nerve, retinal artery, retinal vein, blepharitis, hordeolum, chalazion, canaliculus, dacryocystitis, corneal abrasion, corneal ulcer, ultraviolet keratitis, herpes keratitis, astigmatism, bacterial conjunctivitis, viral conjunctivitis, episcleritis, globe rupture, iritis, uveitis, anterior uveitis, posterior uveitis, hypopyon, hyphema, acute angle closure glaucoma, congenital pupillary deformity, coloboma, globe rupture, nevus, essential iris atrophy, cataracts, presbyopia, myopia, hyperopia, traumatic iritis, iridocyclitis, ciliary body melanoma, vitreous degeneration, vitreous hemorrhage, endophthalmitis, macular degeneration, retinal detachment, choroid nevus, choroid detachment, papilledema, optic nerve glioma, optic nerve meningioma, anterior ischemic optic neuropathy, retinal artery occlusion, retinal vein occlusion.

## USER GUIDE

[Table t1-jetem-5-3-t42]

**Table t1-jetem-5-3-t42:** 

List of Resources:	
Abstract	42
User Guide	44
Learner Materials	48
iRAT	48
gRAT	51
GAE	55
Instructor Materials	56
RAT Key	57
GAE Key	61
Brief Wrap Up	62

**Learner Audience:**

Medical students, interns, junior residents, senior residents

**Time Required for Implementation:**

The instructor will likely require 1–2 hours depending on comfort with the material. Learners should expect to spend approximately 30 minutes to one hour for pre-reading. The recommended time for the in-class segment is two hours; however, this can be flexible depending on material covered and time spent on review after the project aspect of the lesson.

**Recommended Number of Learners per Instructor:**

Approximately 20–45 learners per instructor would be reasonable for this activity given that the instructor does not need to provide a significant amount of one-on-one instruction. Additional instructors would enhance one-on-one teaching during the building phase of the implementation.

**Topics:**

Eye lid, tear duct, cornea, conjunctiva, pupil, iris, lens, anterior chamber, vitreous body, posterior chamber, retina, macula, choroid, optic disc, optic nerve, retinal artery, retinal vein, blepharitis, hordeolum, chalazion, canaliculus, dacryocystitis, corneal abrasion, corneal ulcer, ultraviolet keratitis, herpes keratitis, astigmatism, bacterial conjunctivitis, viral conjunctivitis, episcleritis, globe rupture, iritis, uveitis, anterior uveitis, posterior uveitis, hypopyon, hyphema, acute angle closure glaucoma, congenital pupillary deformity, coloboma, globe rupture, nevus, essential iris atrophy, cataracts, presbyopia, myopia, hyperopia, traumatic iritis, iridocyclitis, ciliary body melanoma, vitreous degeneration, vitreous hemorrhage, endophthalmitis, macular degeneration, retinal detachment, choroid nevus, choroid detachment, papilledema, optic nerve glioma, optic nerve meningioma, anterior ischemic optic neuropathy, retinal artery occlusion, retinal vein occlusion.

**Objectives:**

By the end of this session the participant will be able to:

1. Describe basic anatomy of the eye.
2. Build a basic model of the eye.
3. Identify which diseases are associated with which parts of the eye.
4. Identify the pathophysiology behind diseases.
5. Name correct treatment for each disease.

### Linked objectives and methods

For the learner responsible content (LRC), students are required to read an article (listed below) on eye pathology and anatomy. The reading provides background for objectives 1 and 3–5.

When students begin the lesson, they will take the individualized readiness assessment test (iRAT) which asks the students to recall information from the LRC. This enhances the spaced repetition of learning concepts (distributed practice). The iRAT focuses on eye pathology, thus covering objectives 3 and 4. The student will then join a group of 4–6 other students who will complete the same test together, the group readiness assessment test (gRAT). They will then have the opportunity to discuss their individual answers with one another, increasing understanding of the basic concepts. After completing the RATs, students will then be given the group application exercise (GAE). The GAE will task the learners with building their own eye using basic household materials (objective 2). The post-GAE wrap up session will review all learning objectives.

### Recommended pre-reading for instructor

The instructor may find the following resources useful prior to teaching; however, instructors are encouraged to seek any resources that help them prepare for their unique knowledge deficits.

1. Long B, Koyfman A. emDocs. Acute visual loss in the emergency department: Pearls and pitfalls. http://www.emdocs.net/8578-2/. Published April 18, 2016. Accessed March 26, 2019.
2. Muth CC. Eye emergencies. *JAMA*. 2017;318(7):676. doi:10.1001/jama.2017.9899.
3. Pokhrel PK, Loftus SA. Ocular emergencies. *Am Fam Physician*. 2007;76(6): 829–36.

### Learner Responsible Content (LRC)

The first assignment was required. The second and third assignments are suggested for supplemental reading.

4. Long B, Koyfman A. emDocs. Acute visual loss in the emergency department: Pearls and pitfalls. http://www.emdocs.net/8578-2/. Published April 18, 2016. Accessed March 26, 2019.
5. Muth CC. Eye emergencies. *JAMA*. 2017;318(7):676. doi:10.1001/jama.2017.9899.
6. Pokhrel PK, Loftus SA. Ocular emergencies. *Am Fam Physician*. 2007;76(6): 829–36.

### Results and tips for successful implementation

This exercise was presented during residency conference for a group of approximately 24 emergency medicine residents and medical students. It is best implemented in a group setting with between one and six learners per group and two to six groups. We did not obtain a direct assessment of learner acquisition of knowledge; however, we were able to informally assess learner knowledge by responses throughout the session as well as by the iRAT and gRAT. Implementation went smoothly, and we did not make further modifications to the didactic session.

The following items should be purchased prior to the session:

1. Styrofoam balls, recommended 6–8 inches in diameter (one per group)
2. Colored felt, recommended colors: red, blue, purple, green, black
3. Colored paper
4. Scissors
5. Glue
6. Aluminum foil
7. Paper clips
8. Markers
9. Pipe cleaners, various colors
10. Clear tape
11. Clear plastic food wrap

The instructor will also need to print (or create an online quiz) one copy of the iRAT per learner and one copy of the gRAT and GAE per 4–6 learners.

The learners should be assigned the LRC (listed below) at least three days prior to the didactic session. Upon entry to the didactic session, learners are given the iRAT. Learners are given 5–10 minutes to complete the iRAT and then another 5–10 minutes for the gRAT after splitting into small groups of 4–6 members. The materials were provided for the learners in our implementation; however, the instructor could also suggest students bring their own supplies if learners would like specific materials. Cost depends on regional differences, existing supplies in the residency, and whether learners bring their own supplies. We spent approximately $30.00 for all of the supplies.

For the GAE, learners “build” an eye using the materials provided. They are asked to include a list of anatomical structures that must be present on the model eye (See Group Application Exercise: Eye Anatomy Worksheet). The construction phase is open-resource or “open book.” An instructor should advise learners that their GAE will be “scored.” The students must also name at least one disease/pathologic finding with symptoms and treatment associated with each structure (See Group Application Exercise: Eye Anatomy Worksheet). Learners lose one point for any structure for which they are unable to name a disease. Learners are given extra points for each additional disease named. The instructor can award ten additional points for best eye model, as judged by a panel of faculty present during the exercise. At the end of the implementation, one winning team is selected to “win” the exercise. (Learners can be given plastic or paper medals). After the exercise, the instructor performs a post-GAE review.

This method of implementation was chosen because many ocular pathologies are associated with specific structures. Structure-based learning may allow students to comprehend the anatomy and physiology behind the disease. In this way, learners are better able to identify which pathologies are present on imaging tests and by physical exam. This may result in more rapid identification of diseases when they present in the clinic or emergency department.

## Supplementary Information



## Figures and Tables

**Table t2-jetem-5-3-t42:** 

**EYE ANATOMY WORKSHEET**	**Group #:_______________**

**Table t3-jetem-5-3-t42:** 

**EYE ANATOMY WORKSHEET**	**Group #:_______________**

**Figure f1-jetem-5-3-t42:**
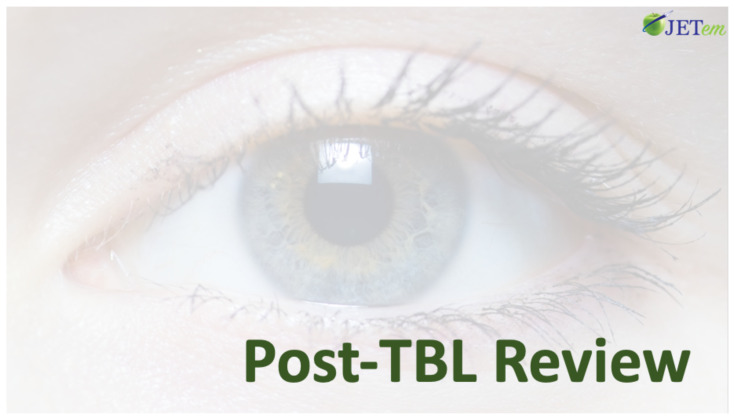


## LEARNER MATERIALS

### Ocular Anatomy: individual Readiness Assessment Test (iRAT)

1. Which chamber(s) of the eye are involved in endophthalmitis?
  - Anterior chamber
  - Posterior chamber
  - Vitreous
  - A&C
  - A, B, & C
2. A 58-year-old male with history of anxiety presents with a hard, painless lump on his eyelid for 14 days. What is the most likely diagnosis?
  - Chalazion
  - Blepharitis
  - Hordeolum
  - Preseptal cellulitis
  - Dacryocystitis
3. A 10-year-old female with history of chronic sinusitis presents with painful eye redness and swelling for one day. The symptoms have progressively worsened and now it hurts when she moves her eyes to look in either direction. What is the most likely diagnosis?
  - Endophthalmitis
  - Preseptal cellulitis
  - Orbital cellulitis
  - Conjunctivitis
  - Uveitis
4. A 28-year-old welder with history of recent cough and congestion presents to the emergency department after he was working in his shop but forgot to wear his mask a few times while welding. A few hours later, he started to notice tearing, erythema, and pain in his eyes. A fluorescein stain shows superficial punctate lesions. What is the most likely cause of his eye pain?
  - Conjunctivitis
  - Ultraviolet keratitis
  - Foreign body
  - Corneal abrasion
  - Viral URI
5. A 21-year-old male presents to the emergency department with eye pain. It is his 21st birthday. He thinks he has had “a few” drinks this evening. His friends tell you that he got into an “epic” bar fight when someone took his “birthday chair.” He is not cooperative with examination but you note that there is a laceration extending from the right mid upper eyelid medially to the nose. He has notable conjunctival injection bilaterally. A fluorescein stain is negative and he has normal ocular pressures. What is the next step in management?
  - Tell him you are going to give his “birthday gurney” to another patient and send him back to the bar after giving him antibiotics.
  - Repair the laceration after a thorough eye wash.
  - Call ophthalmology to repair the laceration.
  - Sedate him and repair the eyelid.
6. The lens bridges the space between which two compartments of the eye?
  - Anterior chamber and posterior chamber
  - Posterior chamber and posterior cavity
  - Anterior cavity and posterior cavity
  - A & B
  - B & C
7. A 26-year-old male with history of type 1 diabetes was playing catcher during a baseball game and was hit in the left eye with a bat as the batter ran for first base. He is not able to cooperate to open his eye well. You think you might have seen a deformed pupil. You note Seidel’s sign on further examination. What is the next step in management?
  - Take intraocular pressures and complete slit lamp examination.
  - Place a rigid eye cover over the eye and call ophthalmology.
  - Prescribe topical antibiotics and discharge home.
  - Obtain CT to assess further.
  - Obtain ultrasound to assess further.

### Ocular Anatomy: group Readiness Assessment Test (gRAT)

Ideally, to make a gRAT using the immediate feedback assessment technique (IF/AT) you may purchase silver “scratch off stickers” (available at amazon.com) and place the stickers over the index letters. If you do not want to create a gRAT in this way, you can simply use the iRAT as a gRAT and either give a blank iRAT copy to each group or have them work together to decide on their final answers.

1. Which anatomic region(s) of the eye is/are involved in endophthalmitis?
  a. Anterior chamber
  b. Posterior chamber
  c. Vitreous
  d. A&C
  • A, B, & C
2. A 58-year-old male with history of anxiety presents with a hard, painless lump on his eyelid for 14 days. What is the most likely diagnosis?
  • Chalazion
  b. Blepharitis
  c. Hordeolum
  d. Preseptal cellulitis
  e. Dacryocystitis
3. A 10-year-old female with history of chronic sinusitis presents with painful, bilateral eye redness and swelling for one day. The symptoms have progressively worsened and now it hurts when she moves her eyes to look in either direction. What is the most likely diagnosis?
  a. Endophthalmitis
  b. Preseptal cellulitis
  • Orbital cellulitis
  d. Conjunctivitis
  e. Uveitis
4. A 28-year-old welder with history of recent cough and congestion presents to the emergency department after he was working in his shop but forgot to wear his mask a few times while welding. A few hours later, he started to notice tearing, erythema, and pain in his eyes. A fluorescein stain shows superficial punctate lesions. What is the most likely cause of his eye pain?
  a. Conjunctivitis
  • Ultraviolet keratitis
  c. Foreign body
  d. Corneal abrasion
  e. Viral URI
5. A 21-year-old male presents to the emergency department with eye pain. It is his 21st birthday. He thinks he has had “a few” drinks this evening. His friends tell you that he got into an “epic” bar fight when someone took his “birthday chair.” He is not cooperative with examination but you note that there is a laceration extending from the right mid upper eyelid medially to the nose. He has notable conjunctival injection bilaterally. A fluorescein stain is negative and he has normal ocular pressures. What is the next step in management?
  a. Tell him you are going to give his “birthday gurney” to another patient and send him back to the bar after giving him antibiotics.
  b. Repair the laceration after a thorough eye wash.
  • Call ophthalmology to repair the laceration.
  d. Sedate him and repair the eyelid.
6. The lens bridges the space between which two compartments of the eye?
  a. Anterior chamber and posterior chamber
  b. Posterior chamber and posterior cavity
  c. Anterior cavity and posterior cavity
  d. A & B
  • B & C
7. A 26-year-old male with history of type 1 diabetes was playing catcher during a baseball game and was hit in the left eye with a bat as the batter ran for first base. He is not able to cooperate to open his eye well. You think you might have seen a deformed pupil. You note Seidel’s sign on further examination. What is the next step in management?
  a. Take intraocular pressures and complete slit lamp examination.
  • Place a rigid eye cover over the eye and call ophthalmology.
  c. Prescribe topical antibiotics and discharge home.
  d. Obtain CT to assess further.
  e. Obtain ultrasound to assess further.

### Ocular Anatomy: Group Application Exercise (GAE) Key

[Table t2-jetem-5-3-t42]

Please include each of these structures in your eye model. Please also include AT LEAST one disease or pathological finding for each structure. You get more points for every disease you list. The team with the best eye model and the most points on their worksheet wins!

1. **Eyelid**
  **_______________________________________**
  **_______________________________________**
  **_______________________________________**
2. **Tear duct**
  **_______________________________________**
  **_______________________________________**
  **_______________________________________**
3. **Cornea**
  **_______________________________________**
  **_______________________________________**
  **_______________________________________**
4. **Conjunctiva**
  **_______________________________________**
  **_______________________________________**
  **_______________________________________**
5. **Pupil**
  **_______________________________________**
  **_______________________________________**
  **_______________________________________**
6. **Iris**
  **_______________________________________**
  **_______________________________________**
  **_______________________________________**
7. **Lens**
  **_______________________________________**
  **_______________________________________**
  **_______________________________________**
8. **Vitreous body**
  **_______________________________________**
  **_______________________________________**
  **_______________________________________**
9. **Anterior Chamber**
  **_______________________________________**
  **_______________________________________**
  **_______________________________________**
10. **Retina**
  **_______________________________________**
  **_______________________________________**
  **_______________________________________**
11. **Macula**
  **_______________________________________**
  **_______________________________________**
  **_______________________________________**
12. **Choroid**
  **_______________________________________**
  **_______________________________________**
  **_______________________________________**
13. **Optic Disc**
  **_______________________________________**
  **_______________________________________**
  **_______________________________________**
14. **Optic nerve**
  **_______________________________________**
  **_______________________________________**
  **_______________________________________**
15. **Retinal artery**
  **_______________________________________**
  **_______________________________________**
  **_______________________________________**
16. **Retinal vein**
  **_______________________________________**
  **_______________________________________**
  **_______________________________________**

## INSTRUCTOR MATERIALS

Answer keys to all exercises with explanations, are on the following pages.

Learners: please do not proceed.

### Ocular Anatomy: Readiness Assessment Test Key (RAT Key)

1. Which anatomic region(s) of the eye is/are involved in endophthalmitis?
  a. Anterior chamber
  b. Posterior chamber
  c. Vitreous
  d. A&C
  • A, B, & C

**Answer:** Endophthalmitis is a serious, inflammatory ocular condition which may cause vision loss. It occurs by multiple modalities including post-traumatic, post-surgical, direct invasion, and hematogenous spread. It may be infectious or sterile. All chambers of the eye (panophthalmitis) can be involved in endophthalmitis. Clinical findings may range from small nodules on the superficial or deep ocular structures to frank purulence of intraocular structures.^7^, ^8^

2. A 58-year-old male with history of anxiety presents with a hard, painless lump on his eyelid for 14 days. What is the most likely diagnosis?
  • Chalazion
  b. Blepharitis
  c. Hordeolum
  d. Preseptal cellulitis
  e. Dacryocystitis

**Answer:** Chalazion is an inflammatory condition of the eyelid which forms due to obstruction of the sebaceous glands in the eyelid. The nodules are usually firm and painless in contrast to hordeolum which is characterized as painful. Chalazia are usually indolent rather than acute. While a hordeolum is usually due to infection, a chalazion is more likely due to chronic, sterile inflammation. Malignancy of the eyelid may sometimes be mistaken for chalazion. Because the question stem names a hard, painless nodule which is subacute, this is most likely chalazion.^9^, ^10^

3. A 10-year-old female with history of chronic sinusitis presents with painful, bilateral eye redness and swelling for one day. The symptoms have progressively worsened and now it hurts when she moves her eyes to look in either direction. What is the most likely diagnosis?
  a. Endophthalmitis
  b. Preseptal cellulitis
  • Orbital cellulitis
  d. Conjunctivitis
  e. Uveitis

**Answer:** Orbital cellulitis is characterized as cellulitis which is posterior to the orbital septum. Patients can present with pain in the affected eye, conjunctival injection, proptosis, fever, chemosis, edema, headache, malaise, and ophthalmoplegia. Development of orbital cellulitis is usually associated with sinusitis, particularly in children. Complications of delayed treatment include vision loss. Treatment is with antibiotics and possible surgical intervention. This patient has ophthalmoplegia and a history of sinusitis making orbital cellulitis the most likely diagnosis.^11^–^13^

4. 28-year-old welder with history of recent cough and congestion presents to the emergency department after he was working in his shop but forgot to wear his mask a few times while welding. A few hours later, he started to notice tearing, erythema, and pain in his eyes. A fluorescein stain shows superficial punctate lesions. What is the most likely cause of his eye pain?
  a. Conjunctivitis
  • Ultraviolet keratitis
  c. Foreign body
  d. Corneal abrasion
  e. Viral URI

**Answer:** Ultraviolet keratitis is damage to the corneal epithelium from ultraviolet light exposure. This type of injury is typically found in welders, skiers/mountaineers (from highly reflective snow), users of tanning booths, or individuals who view the Sun or a solar eclipse directly. Patients may present with pain, tearing, and photophobia. Diagnosis is based on history and detection of superficial punctate lesions on the corneal epithelium. Treatment is eye irrigation, cycloplegic drops, and ocular lubrication. The patient in this prompt is a welder who did not use eye protection with superficial punctate lesions on fluorescein staining, making ultraviolet keratitis most likely.^14^, ^15^

5. A 21-year-old male presents to the emergency department with eye pain. It is his 21st birthday. He thinks he has had “a few” drinks this evening. His friends tell you that he got into an “epic” bar fight when someone took his “birthday chair.” He is not cooperative with examination but you note that there is a laceration extending from the right mid upper eyelid medially to the nose. He has notable conjunctival injection bilaterally. A fluorescein stain is negative and he has normal ocular pressures. What is the next step in management?
  a. Tell him you are going to give his “birthday gurney” to another patient and send him back to the bar after giving him antibiotics.
  b. Repair the laceration after a thorough eye wash.
  • Call ophthalmology to repair the laceration.
  d. Sedate him and repair the eyelid.

**Answer:** This patient has a canalicular laceration. The canalicular system is responsible for draining tears from the eye. It is located medially on the upper and lower eyelids, extending to the nasolacrimal duct and draining into the nose. Injuries to the canalicular apparatus can lead to long-term morbidity due to inability to drain tears properly. Ophthalmology should be utilized for repairs of the ductal system. Repair should not be attempted by emergency medicine practitioners given high rate of complication. Use of microscopy tools improves success of repair.^16^, ^17^

6. The lens bridges the space between which two compartments of the eye?
  a. Anterior chamber and posterior chamber
  b. Posterior chamber and posterior cavity
  c. Anterior cavity and posterior cavity
  d. A & B
  • B & C

**Answer:** The eye is divided into two chambers and two cavities. The anterior cavity is anterior to the lens and composed of the anterior and posterior chambers. The posterior cavity is posterior to the lens (vitreous). The anterior chamber includes structures anterior to the lens and the posterior chamber includes structures between the iris and lens (posterior to the iris). Therefore, the lens divides the posterior chamber and posterior cavity as well as the anterior and posterior cavities.^18^–^19^

7. A 26-year-old male with history of type 1 diabetes was playing catcher during a baseball game and was hit in the left eye with a bat as the batter ran for first base. He is not able to cooperate to open his eye well. You think you might have seen a deformed pupil. You note Seidel’s sign on further examination. What is the next step in management?=
  a. Take intraocular pressures and complete slit lamp examination.
  • Place a rigid eye cover over the eye and call ophthalmology.
  c. Prescribe topical antibiotics and discharge home.
  d. Obtain CT to assess further.
  e. Obtain ultrasound to assess further.

**Answer:** The patient in the prompt likely has a traumatic globe rupture. This is evidenced by the mechanism and history, physical exam findings suggesting deformed pupil, and positive Seidel’s sign. Seidel’s sign is the seeping of fluorescein from the site of injury suggesting leakage of the aqueous fluid. Globe rupture is characterized by disruption of the cornea, sclera, or both and is an ophthalmologic emergency. Ophthalmology must be consulted immediately for operative repair.^20^–^22^

### Ocular Anatomy: Group Application Exercise (GAE) Key

[Table t3-jetem-5-3-t42]

Please include each of these structures in your eye model. Please also include AT LEAST one disease or pathological finding for each structure. You get more points for every disease you list. The team with the best eye model and the most points on their worksheet wins!

1. **Eyelid**
  Blepharitis
  Hordeolum
  Chalazion
2. **Tear duct**
  Laceration
  Dacryocystitis
3. **Cornea**
  Corneal abrasions/ulcer
  UV keratitis
  Astigmatism
4. **Conjunctiva**
  Bacterial conjunctivitis
  Viral conjunctivitis
5. **Pupil**
  Congenital pupillary deformity
  Coloboma
  Globe rupture
6. **Iris**
  Nevus
  Coloboma
  Essential iris atrophy
7. **Lens**
  Cataracts
  Presbyopia
  Myopia/hyperopia
8. **Vitreous body**
  Vitreous degeneration
  Vitreous hemorrhage
  Endophthalmitis
9. **Anterior Chamber**
  Uveitis/iritis
  Hypopyon/hyphema
  Acute angle glaucoma
10. **Retina**
  Retinal detachment
11. **Macula**
  Macular degeneration
12. **Choroid**
  Choroid nevus
  Choroid melanoma
  Choroid detachment
13. **Optic Disc**
  Papilledema
14. **Optic nerve**
  Optic nerve glioma
  Optic nerve meningioma
  Anterior ischemic optic neuropathy
15. **Retinal artery**
  Retinal artery occlusion
16. **Retinal vein**
  Retinal vein occlusion

### Ocular Anatomy: Brief Wrap Up (optional)

Please see the attached PowerPoint presentation for an example of a wrap up presentation. The presentation can be modified based on time constraints and instructor preference. We recommend reviewing the major ocular emergencies and nonemergency pathologies that one might encounter in the clinic or emergency department. Review of this PowerPoint took one hour for our program, allowing time for discussion. We feel that this was a valuable use of time for our residency program.[Fig f1-jetem-5-3-t42]
