# Supplementary material for: Build Your Own Eye: A Method for Teaching Ocular Anatomy and Pathophysiology
Source: J Educ Teach Emerg Med. 2020 Jul 15;5(3):T42–62. doi: 10.21980/J8GS8W (PMC10332548; doi:10.21980/J8GS8W)
Supplement: Supplementary file 1 [file jetem-5-3-t42-supp1.pptx]

## Slide 1
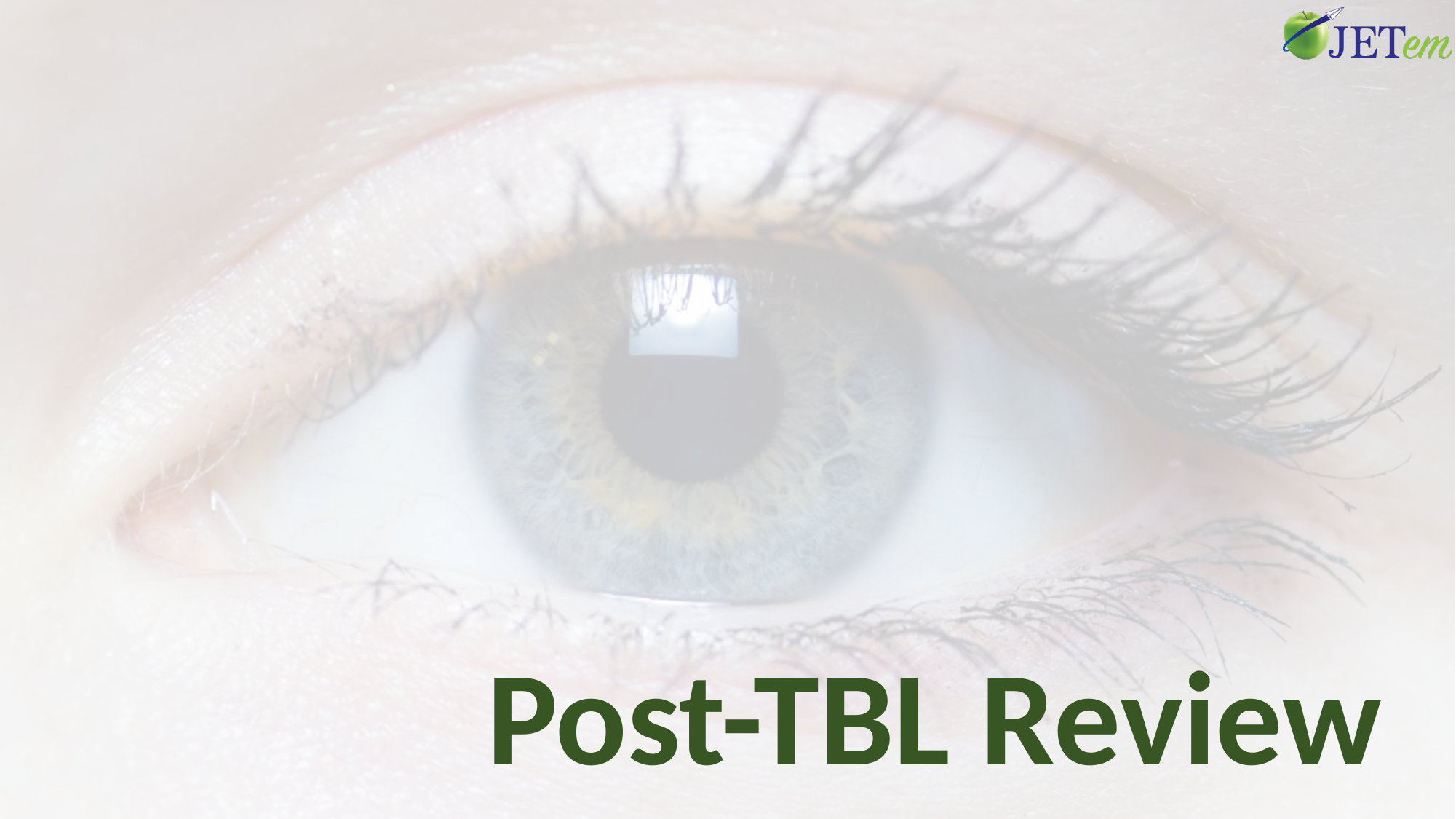

Post-TBL Review

## Slide 2
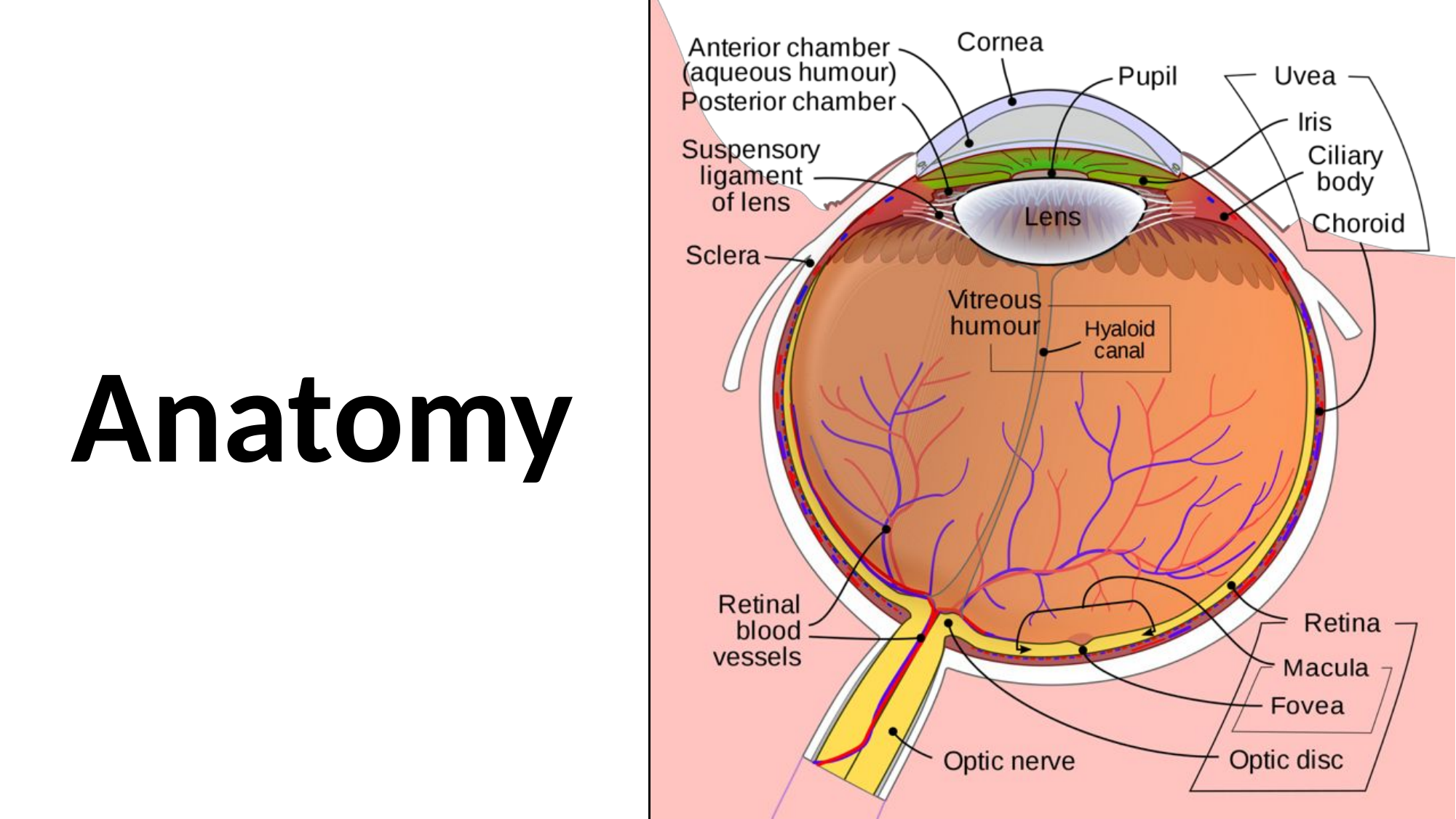

Anatomy

## Slide 3
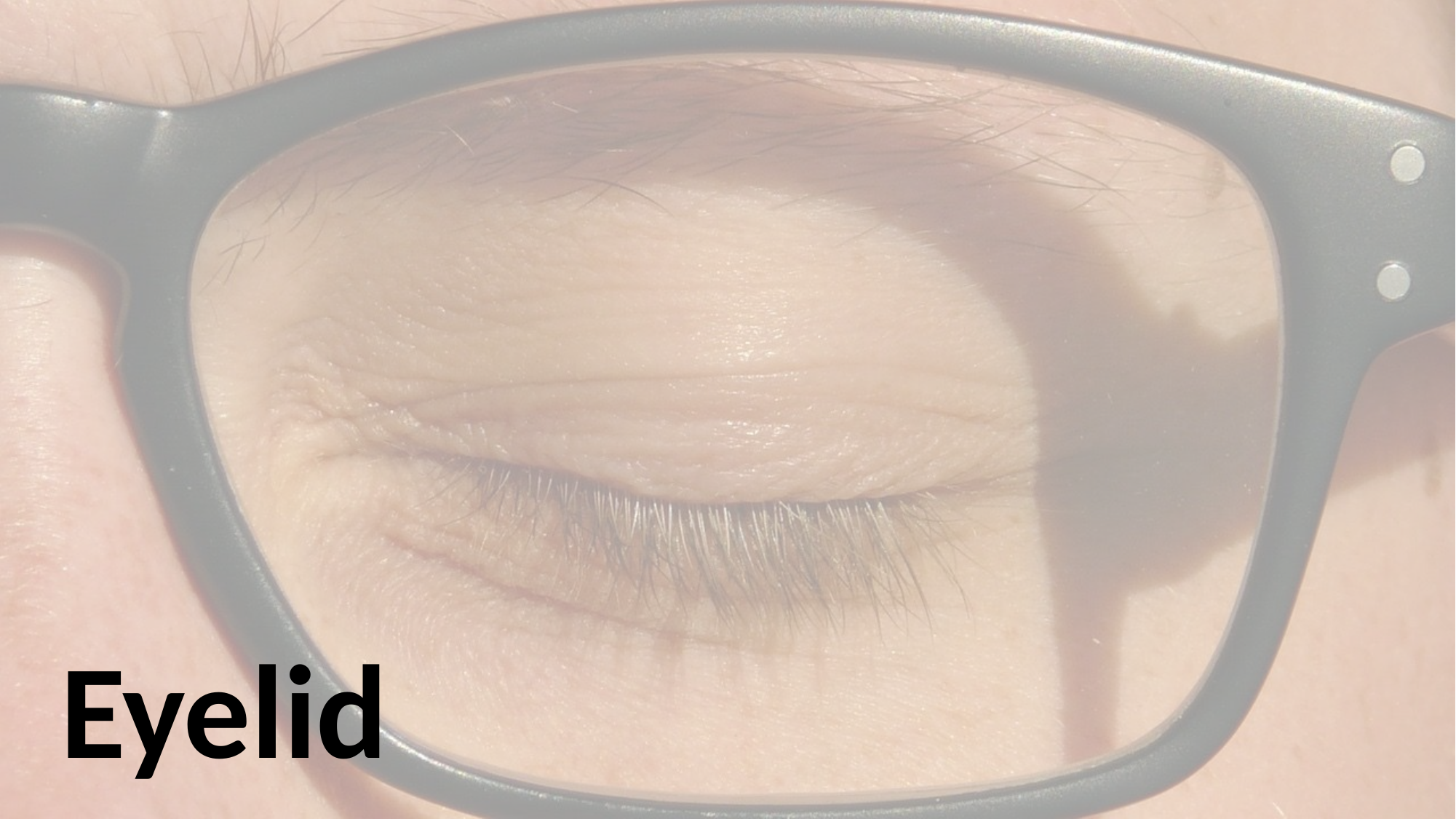

Eyelid

## Slide 4
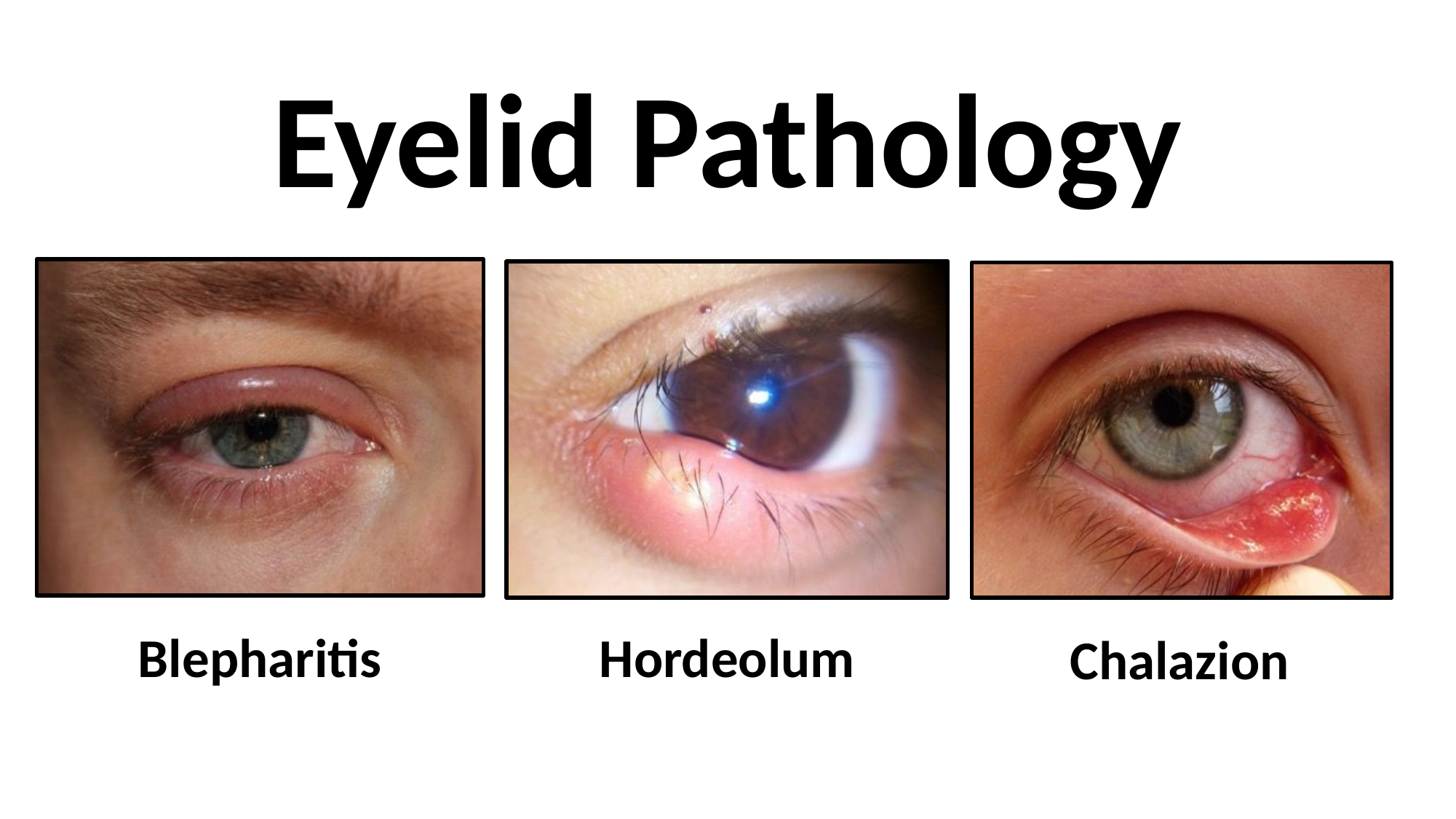

Eyelid Pathology
Blepharitis
Hordeolum
Chalazion

## Slide 5
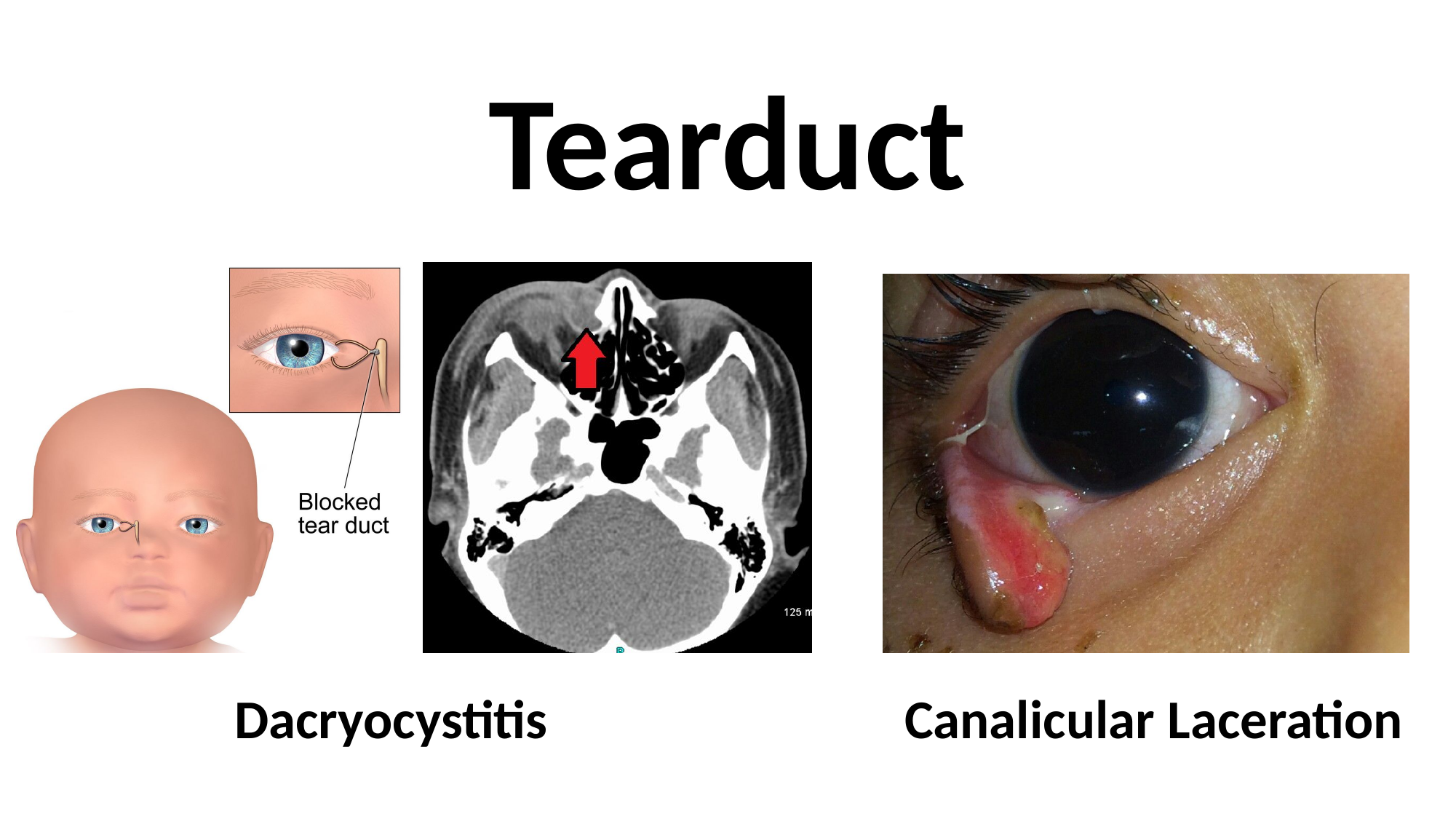

Tearduct
Dacryocystitis
Canalicular Laceration

## Slide 6
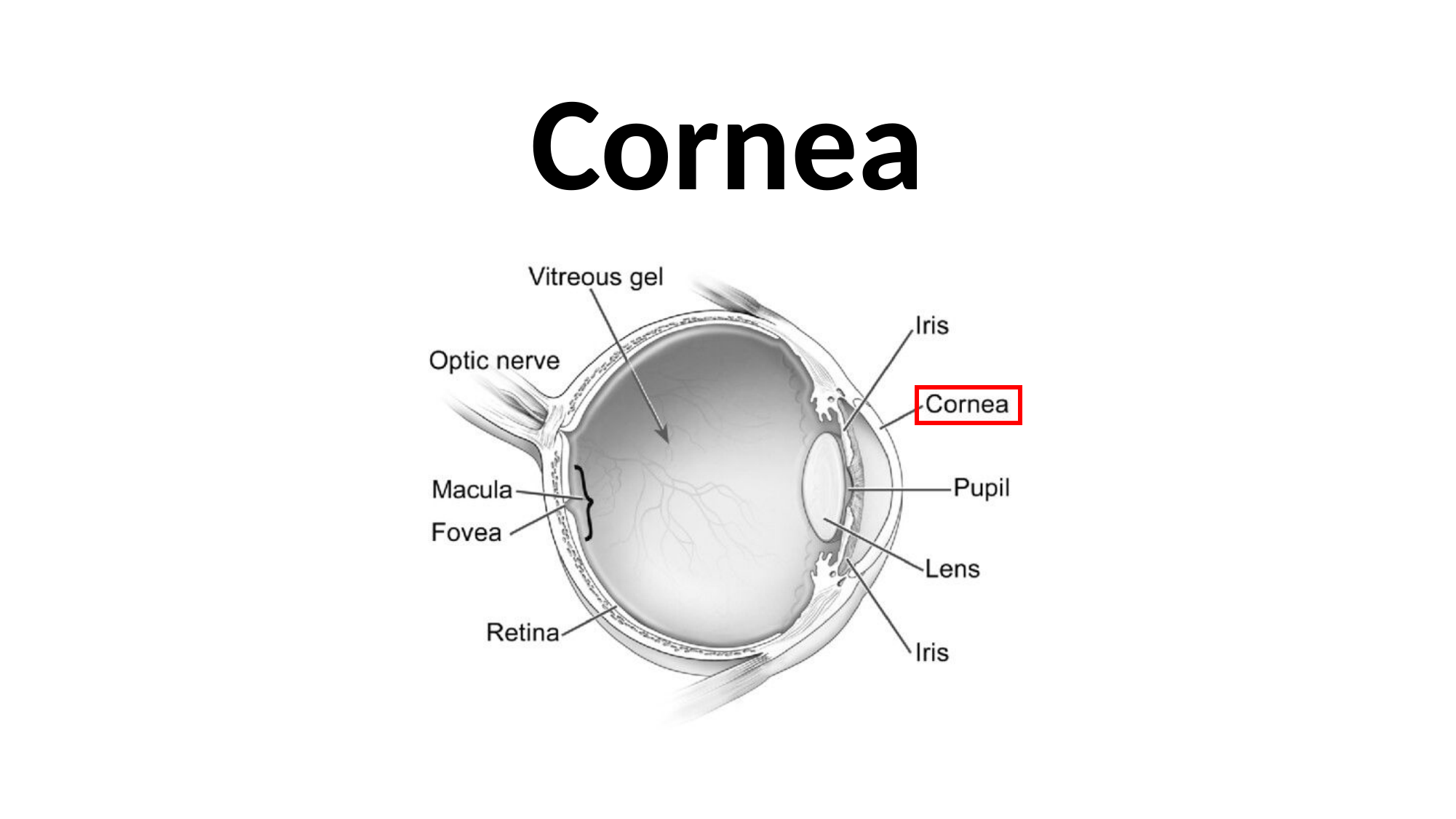

Cornea

## Slide 7
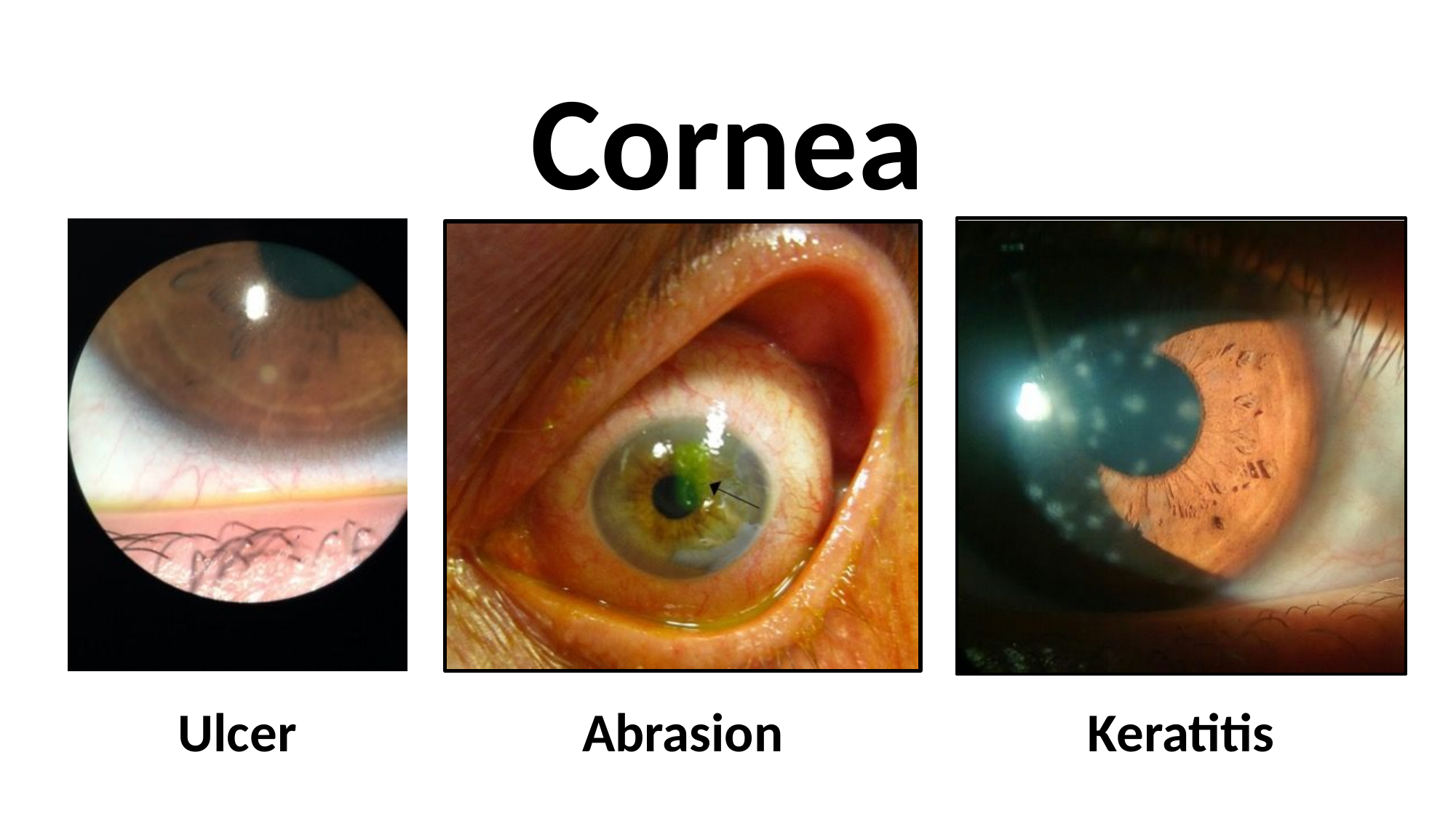

Cornea
Ulcer
Abrasion
Keratitis

## Slide 8
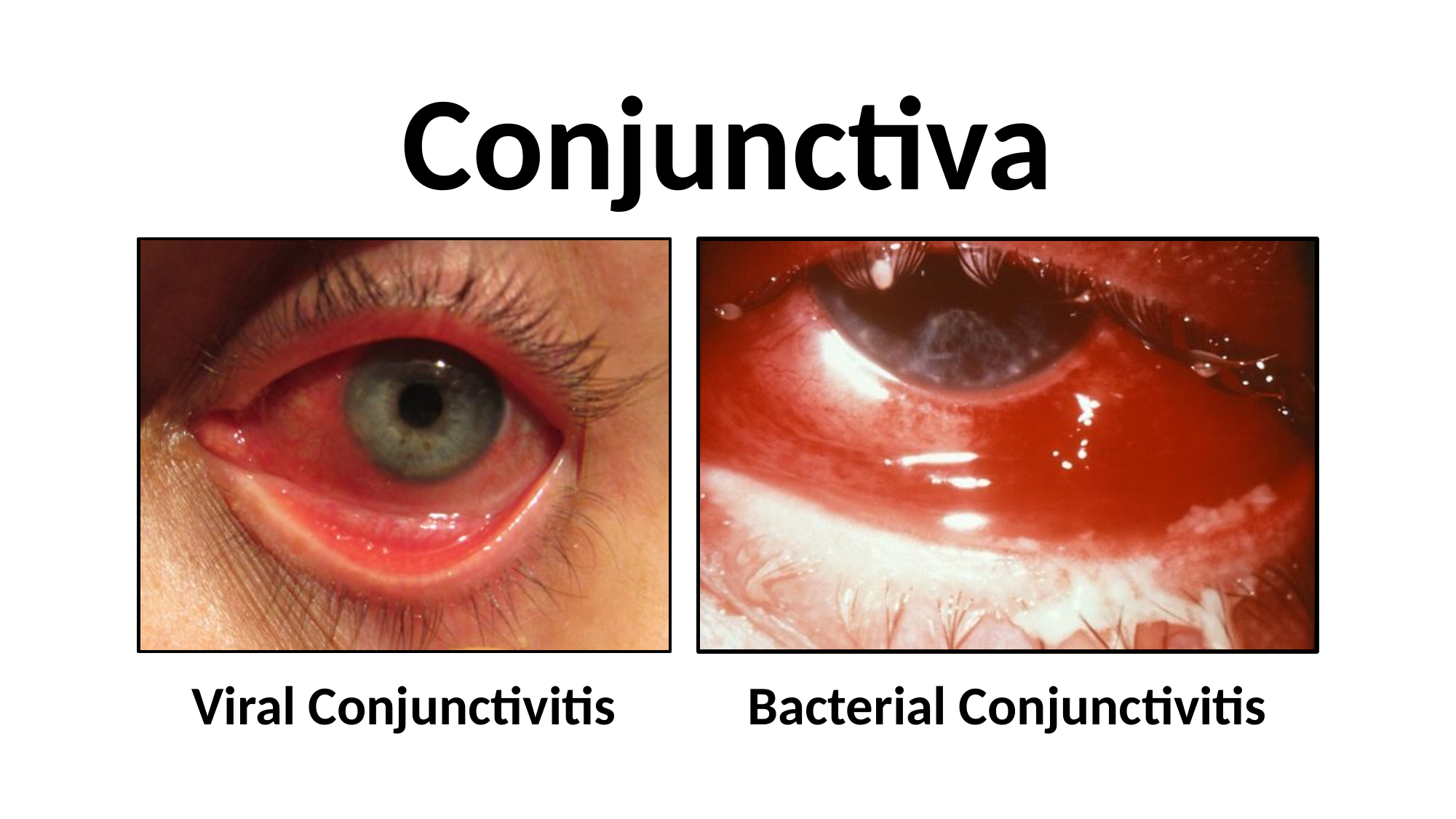

Conjunctiva
Viral Conjunctivitis
Bacterial Conjunctivitis

## Slide 9
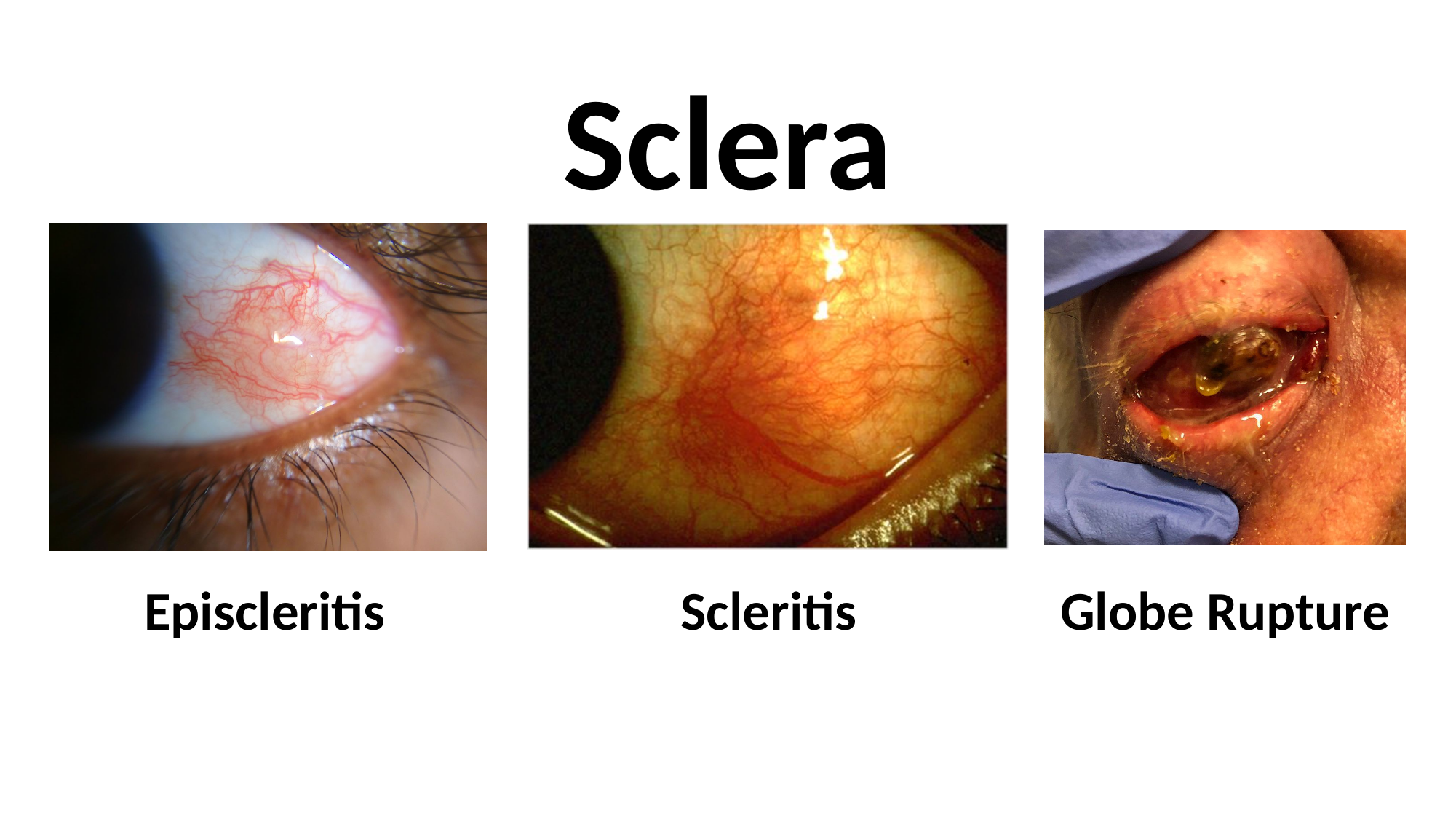

Sclera
Episcleritis
Scleritis
Globe Rupture

## Slide 10
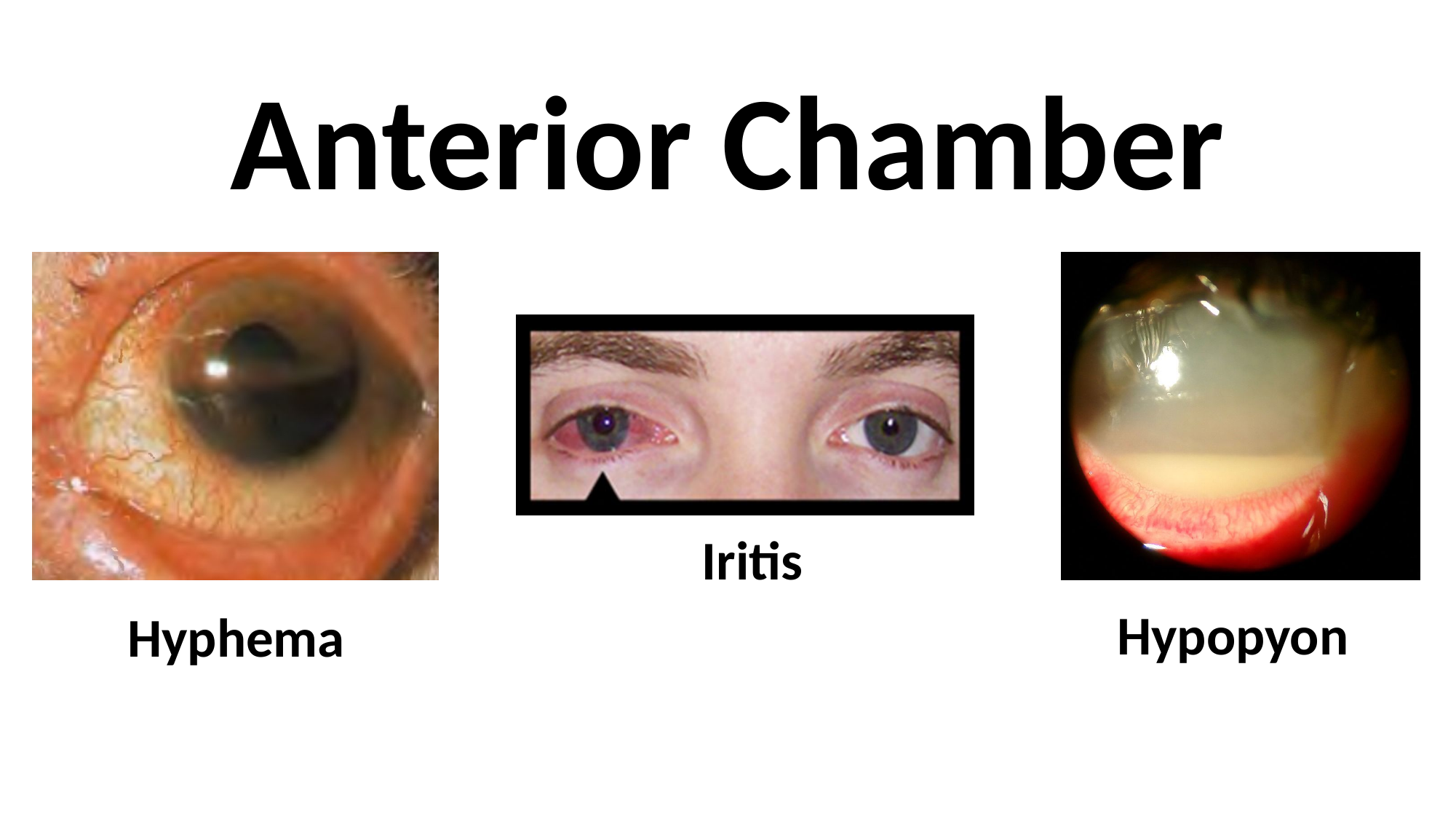

Anterior Chamber
Iritis
Hypopyon
Hyphema

## Slide 11
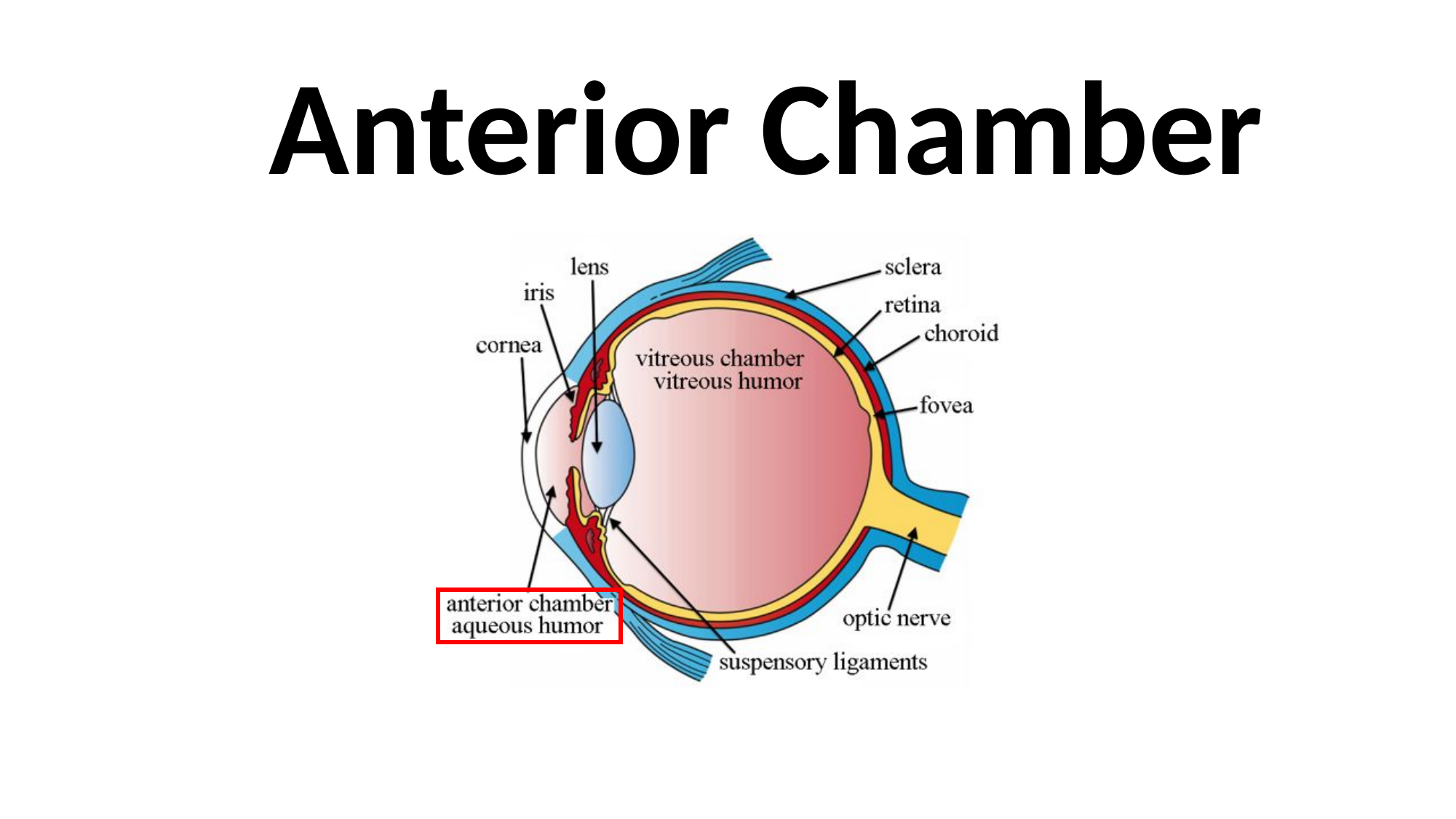

Anterior Chamber

## Slide 12
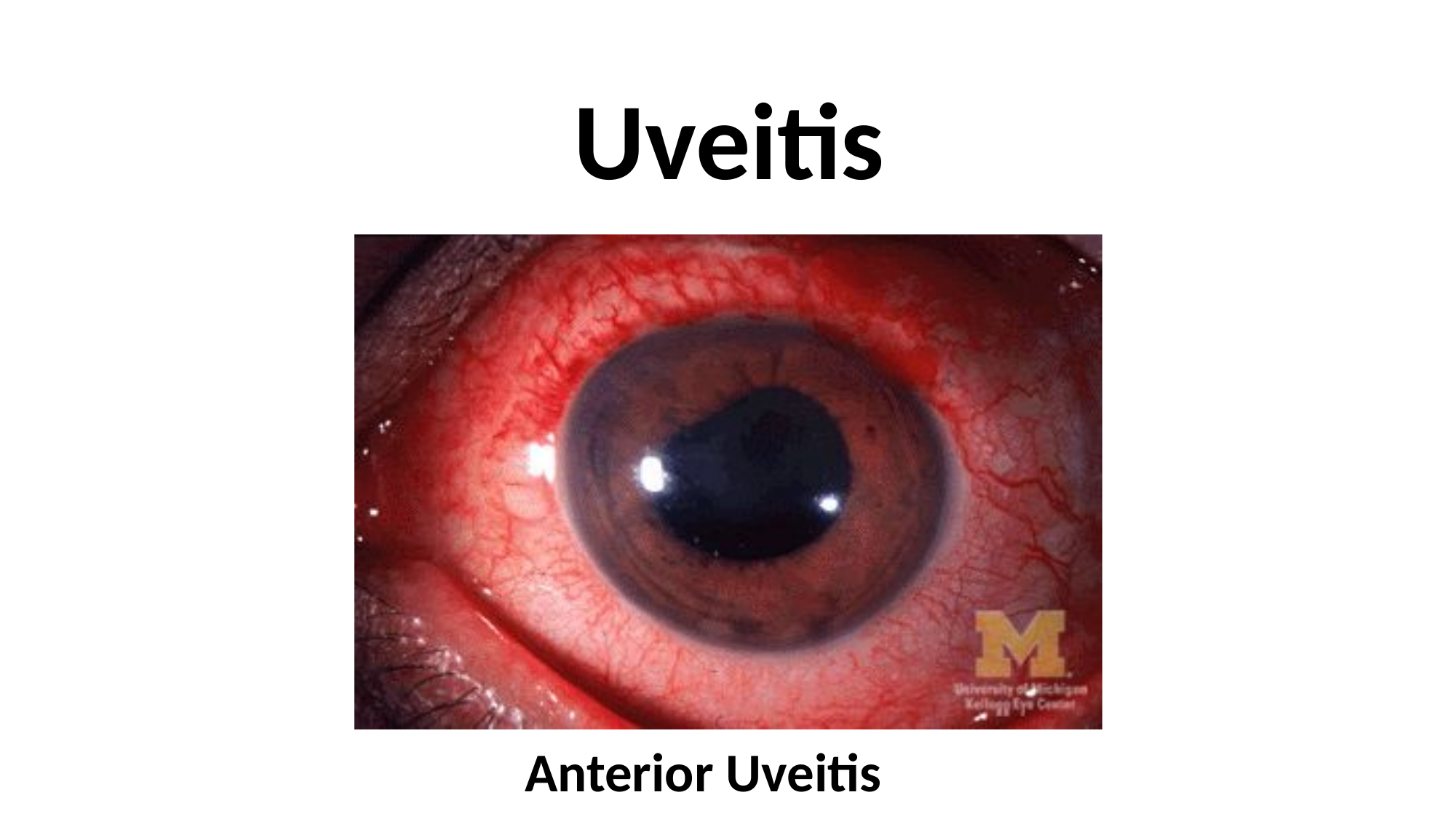

Uveitis
Anterior Uveitis

## Slide 13
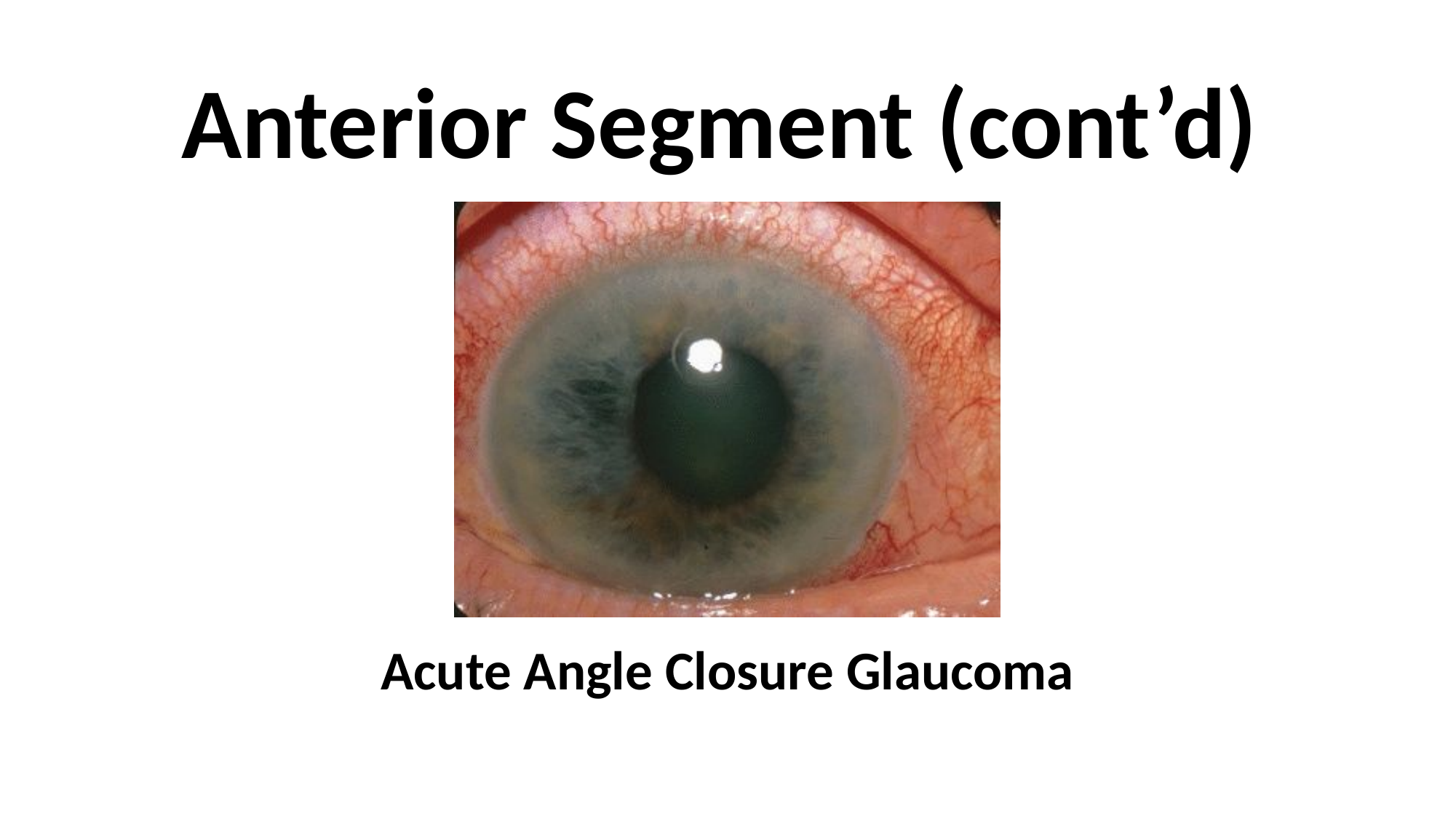

Anterior Segment (cont’d)
Acute Angle Closure Glaucoma

## Slide 14
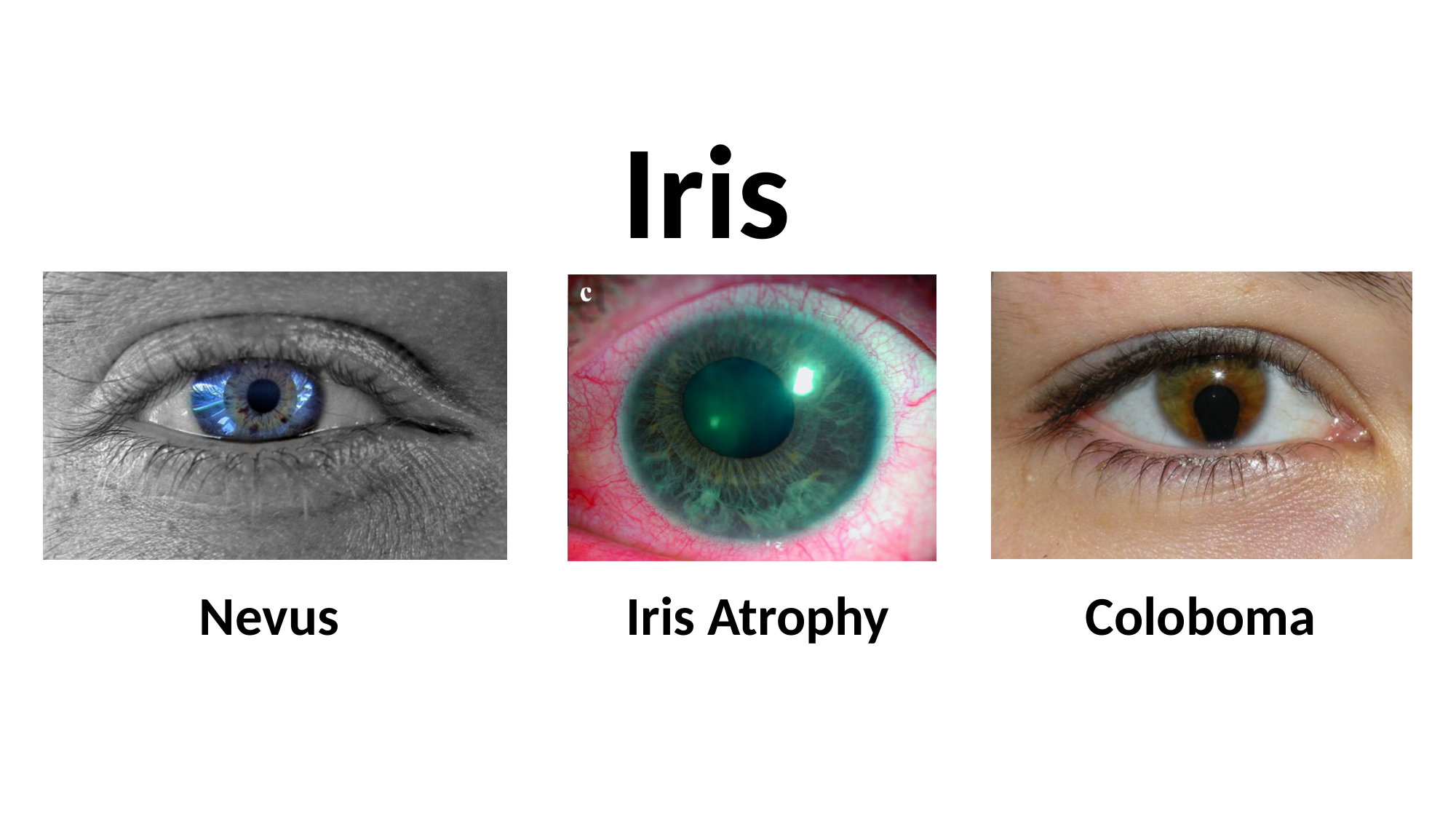

Iris
Nevus
Iris Atrophy
Coloboma

## Slide 15
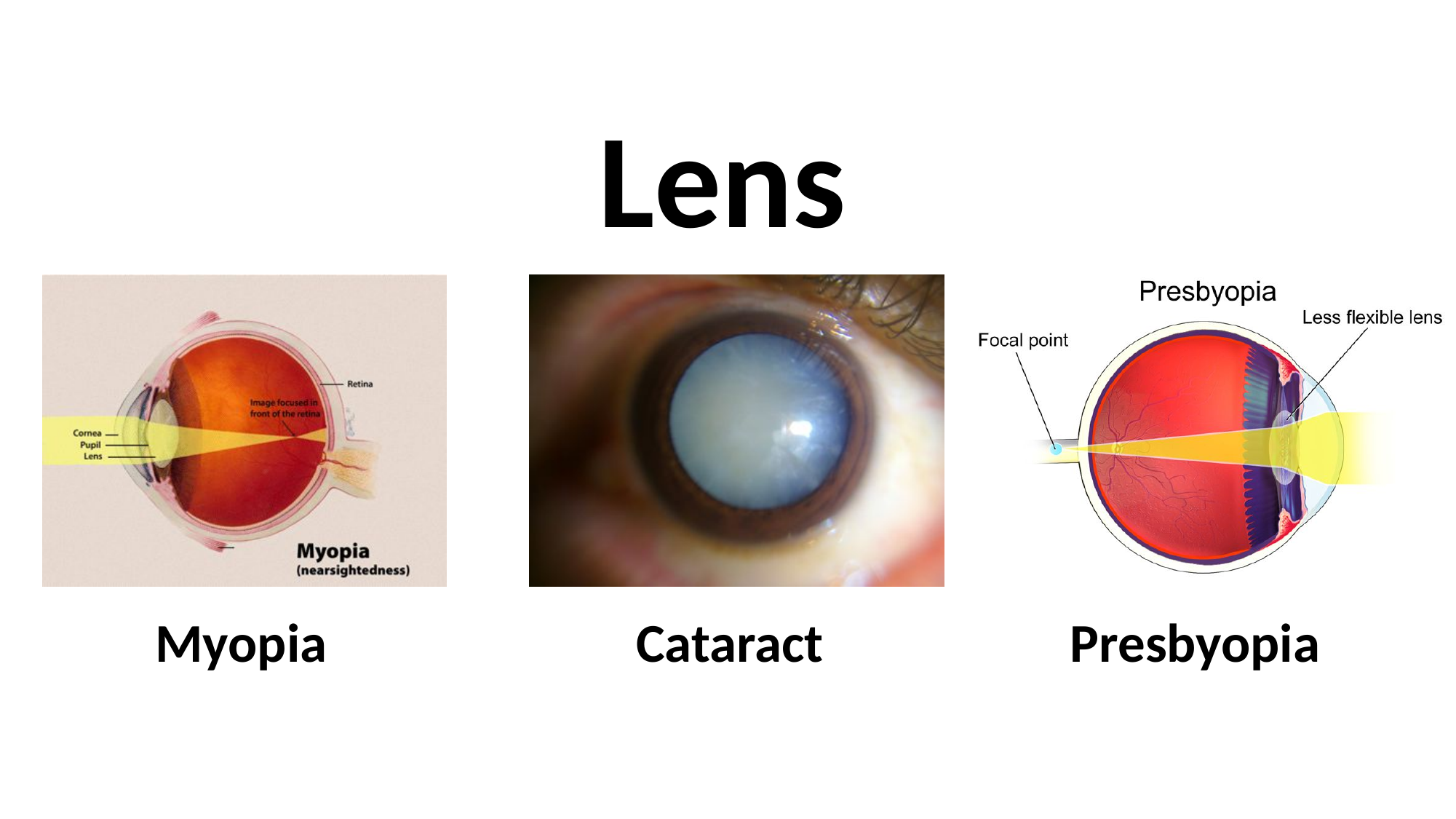

Lens
Myopia
Cataract
Presbyopia

## Slide 16
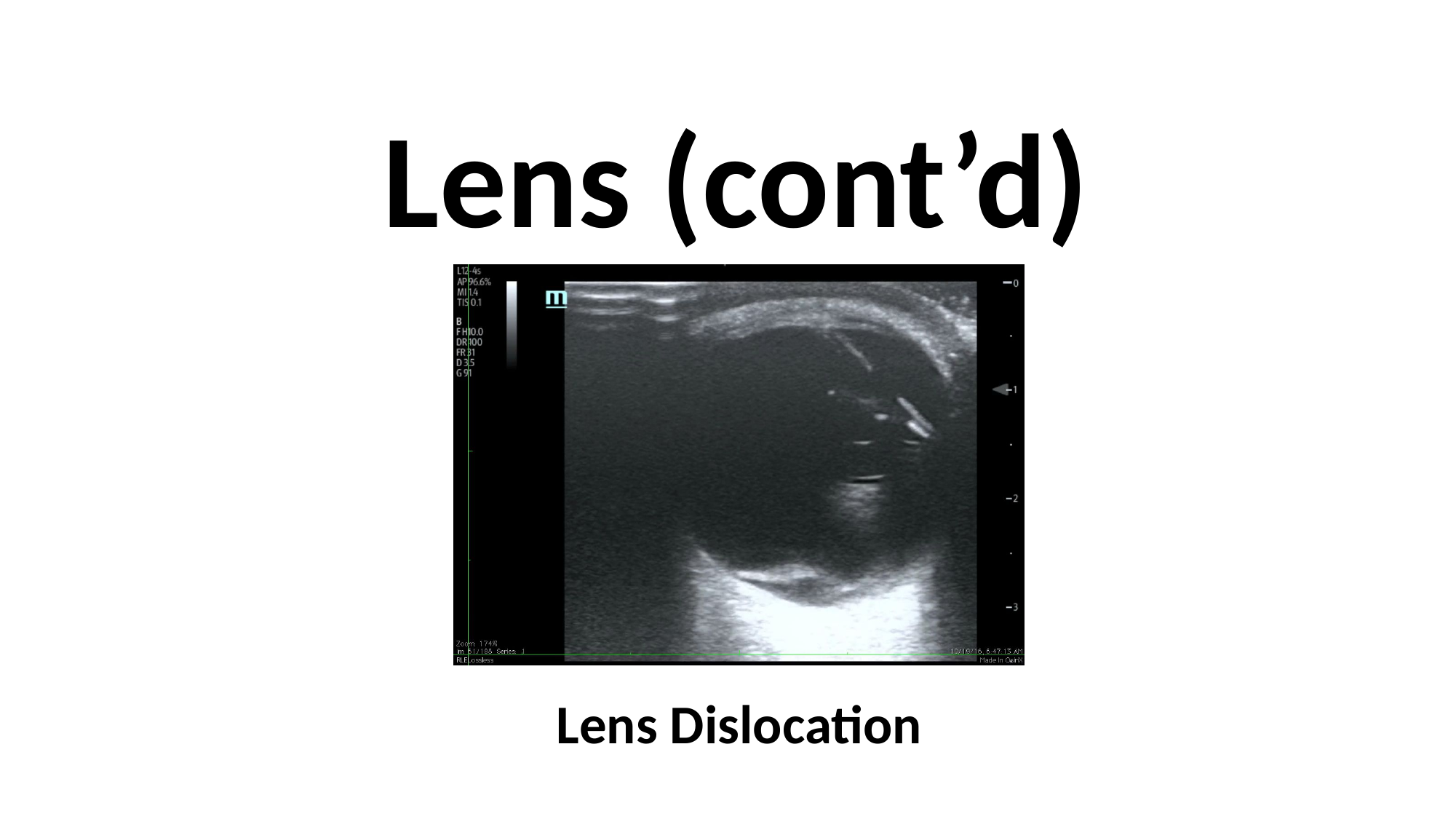

Lens (cont’d)
Lens Dislocation

## Slide 17
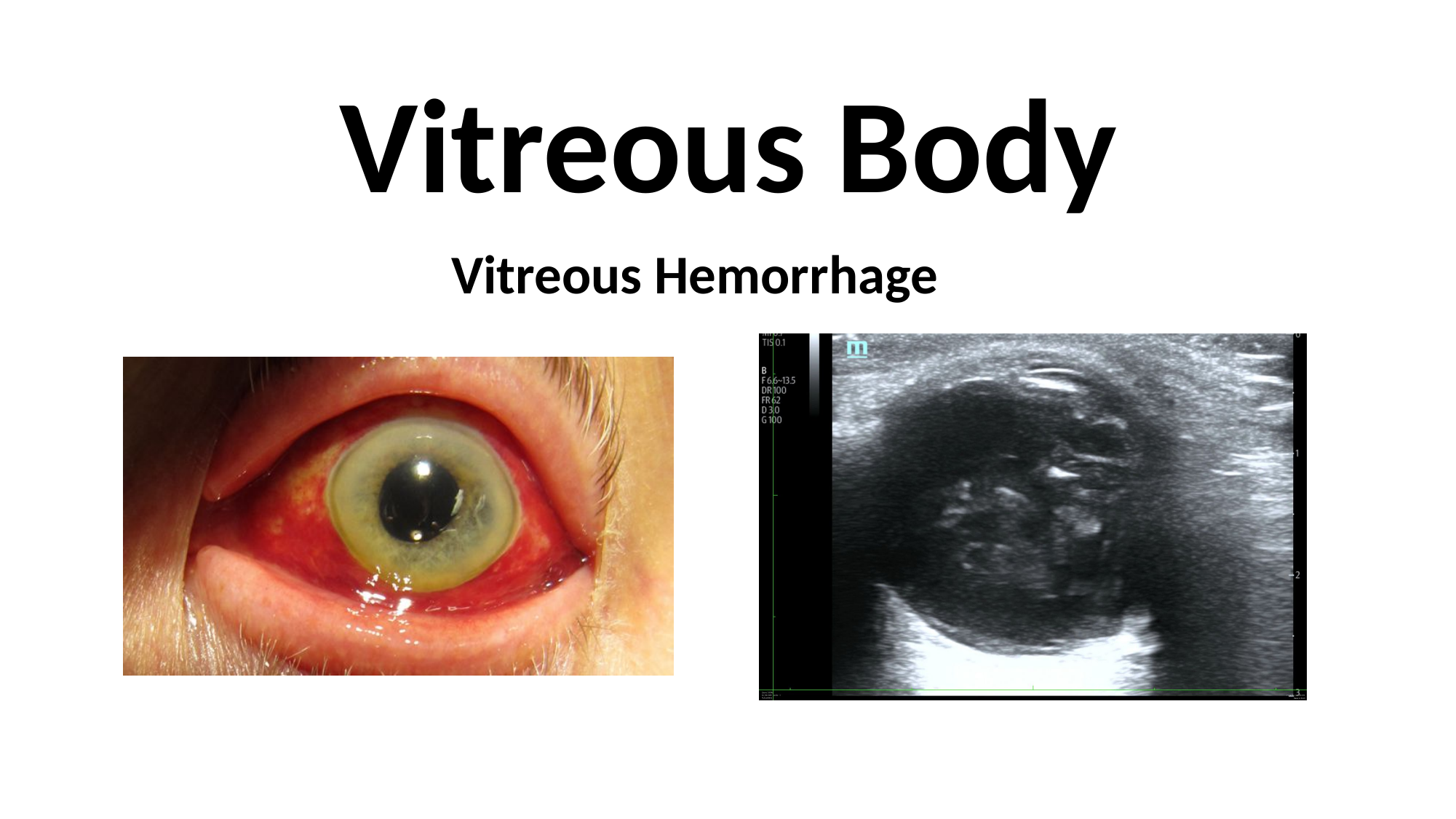

Vitreous Body
Vitreous Hemorrhage

## Slide 18
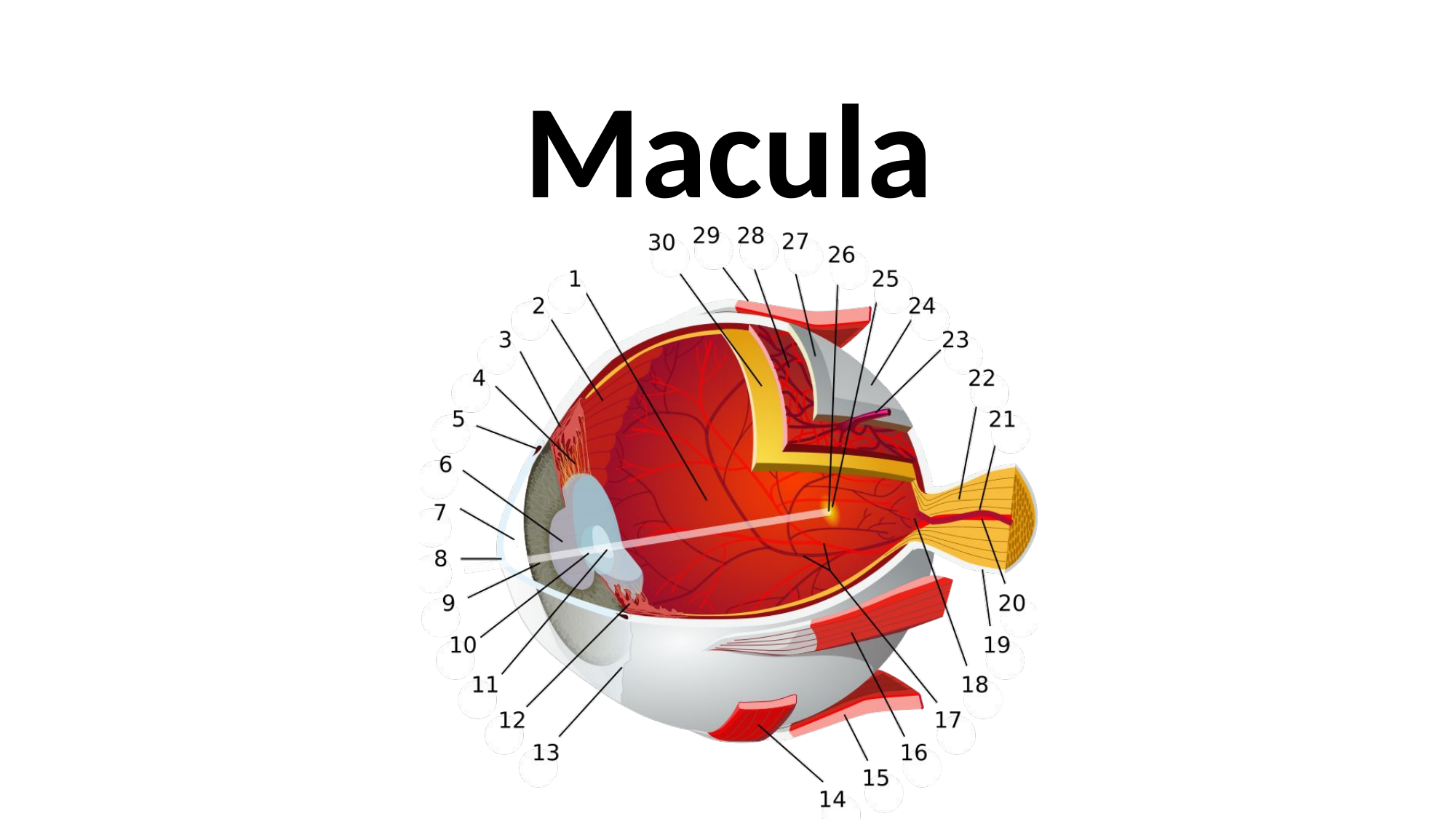

Macula

## Slide 19
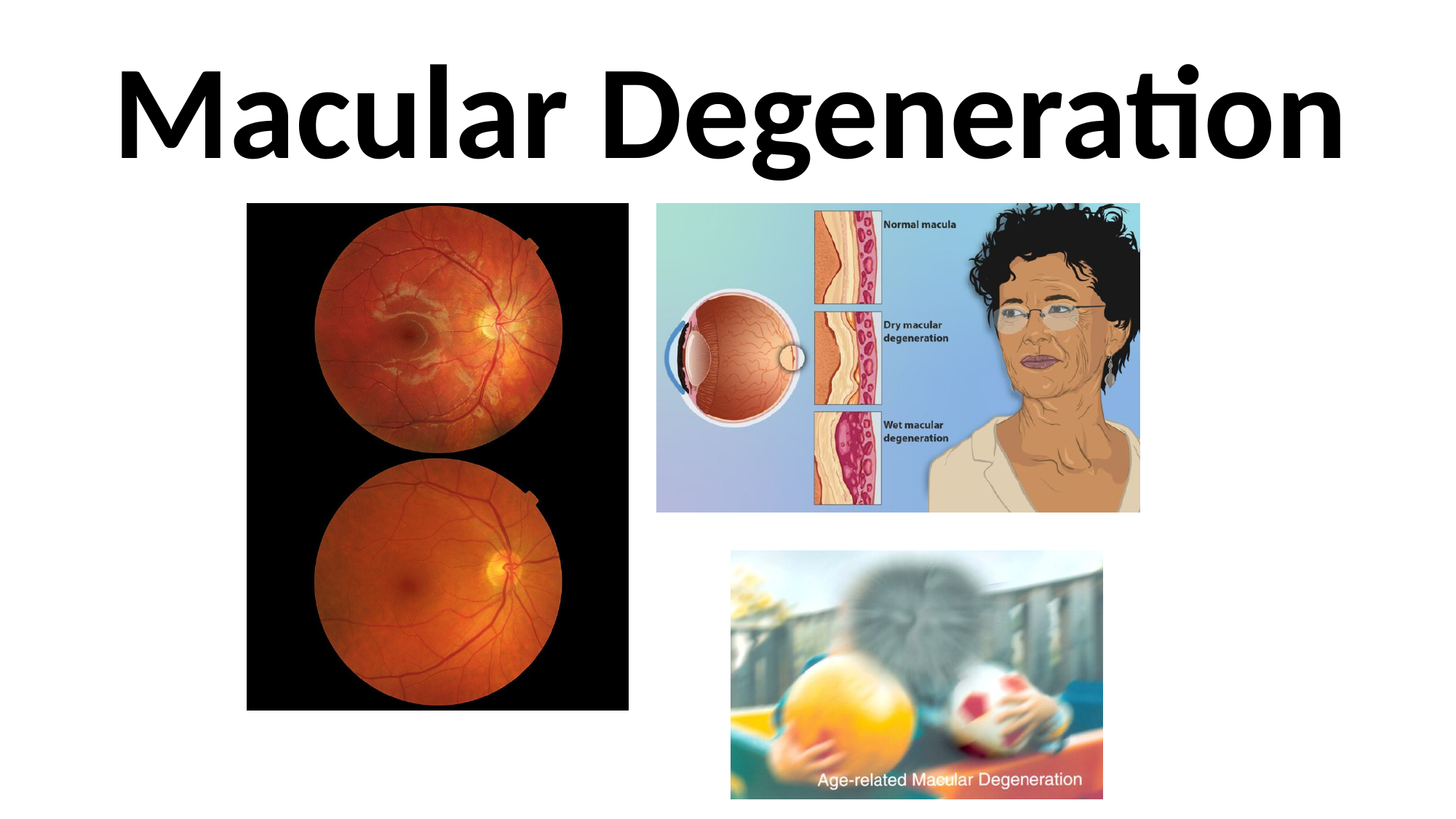

Macular Degeneration

## Slide 20
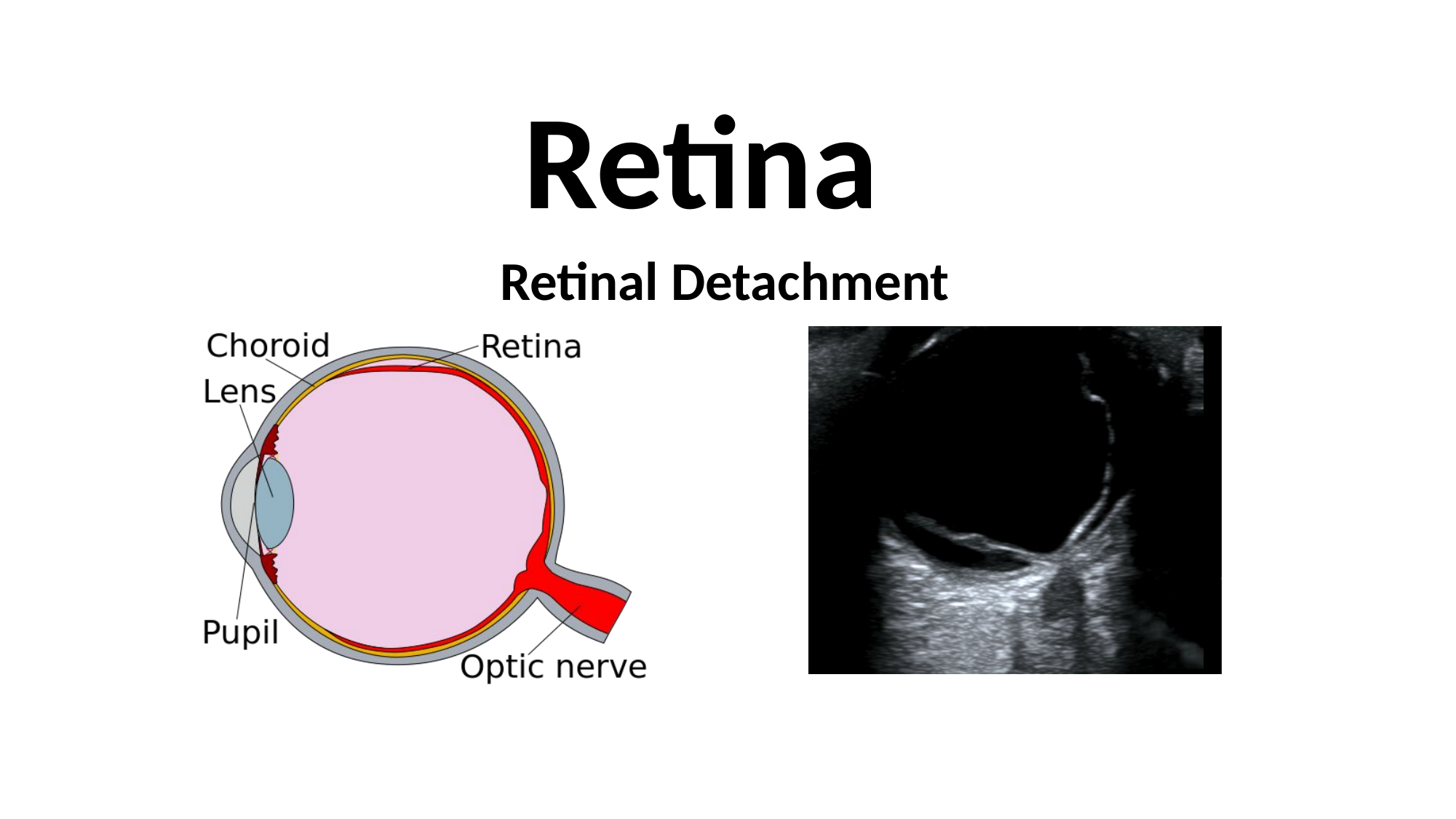

Retina
Retinal Detachment

## Slide 21
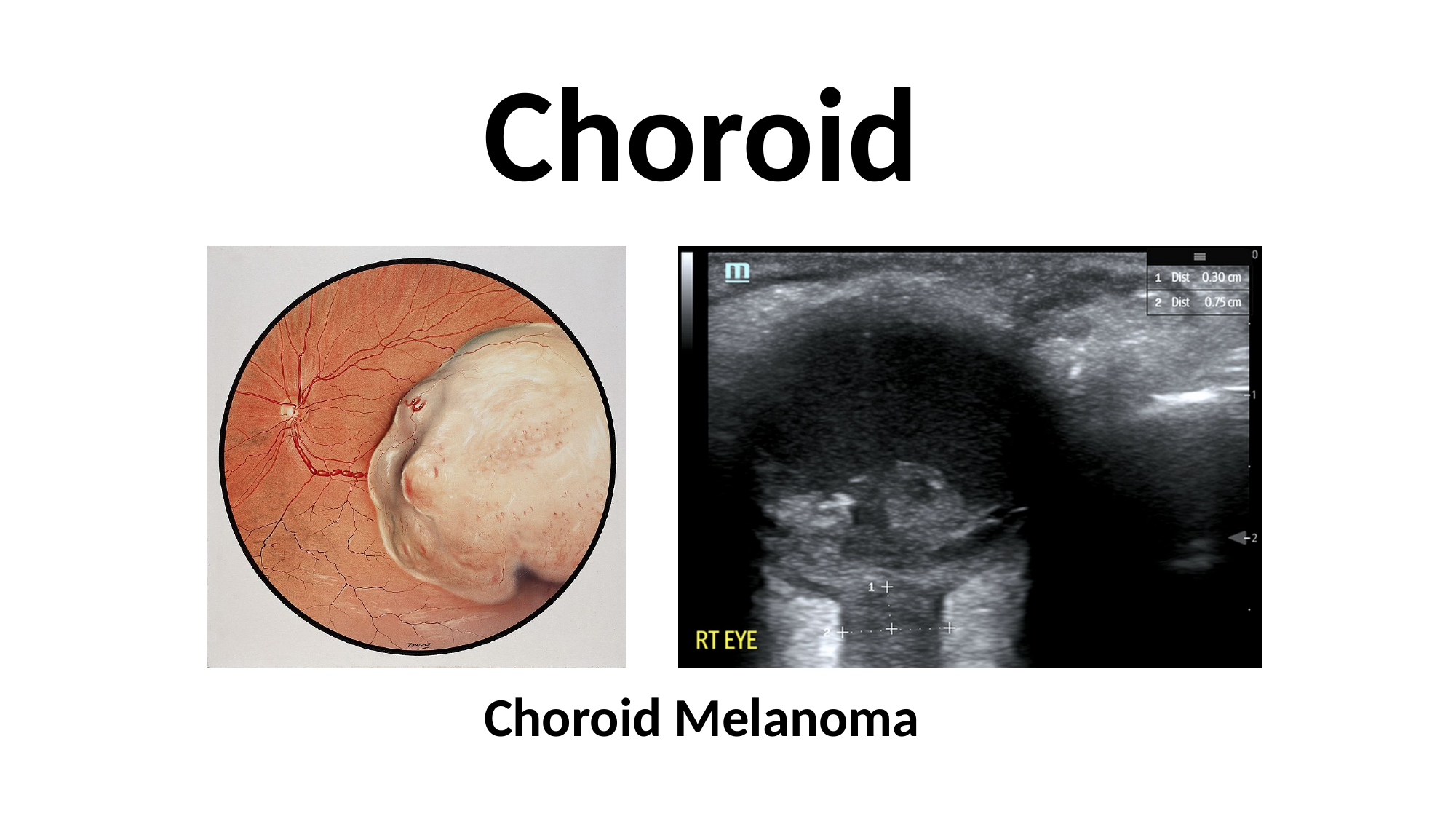

Choroid
Choroid Melanoma

## Slide 22
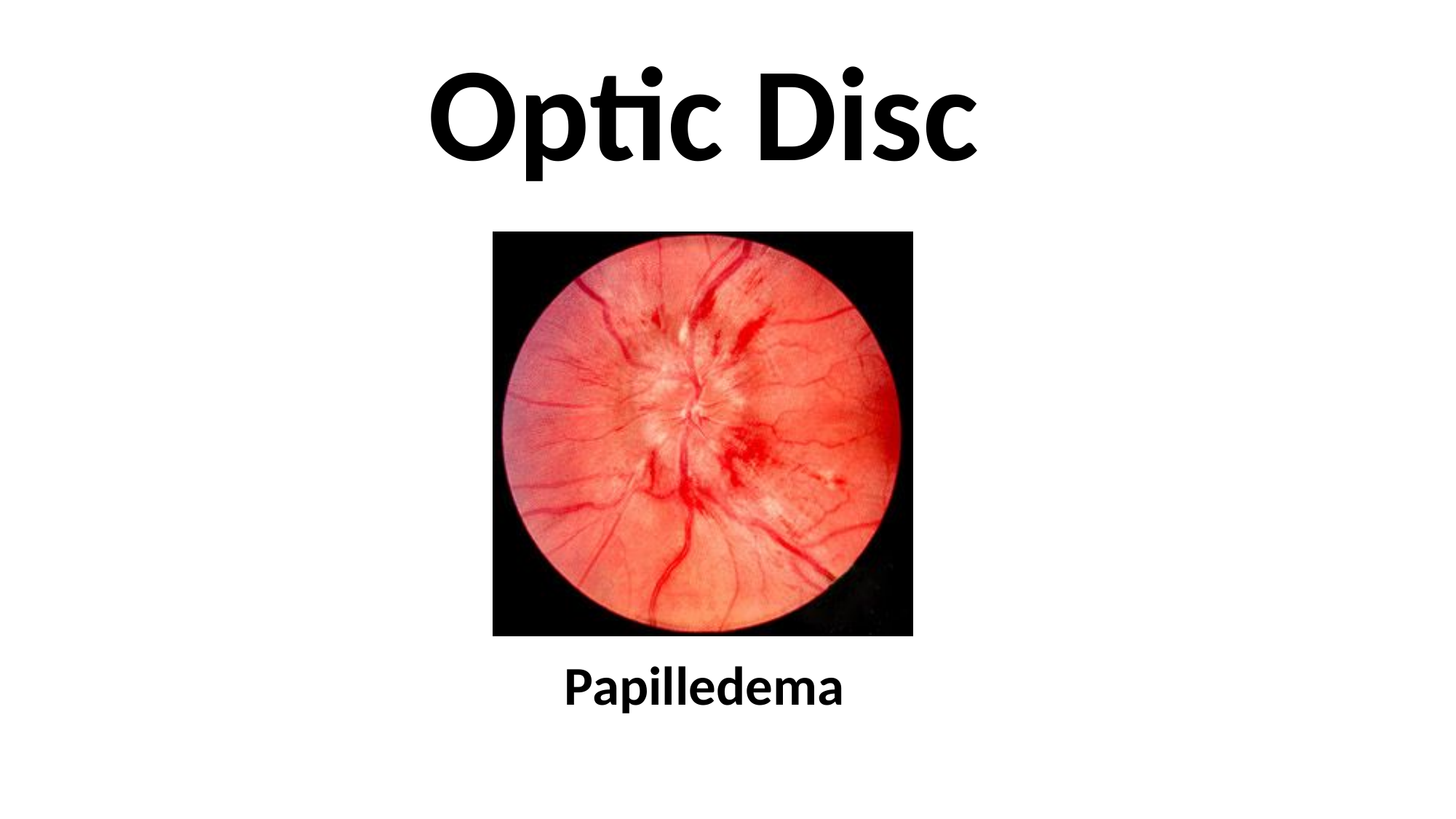

Optic Disc
Papilledema

## Slide 23
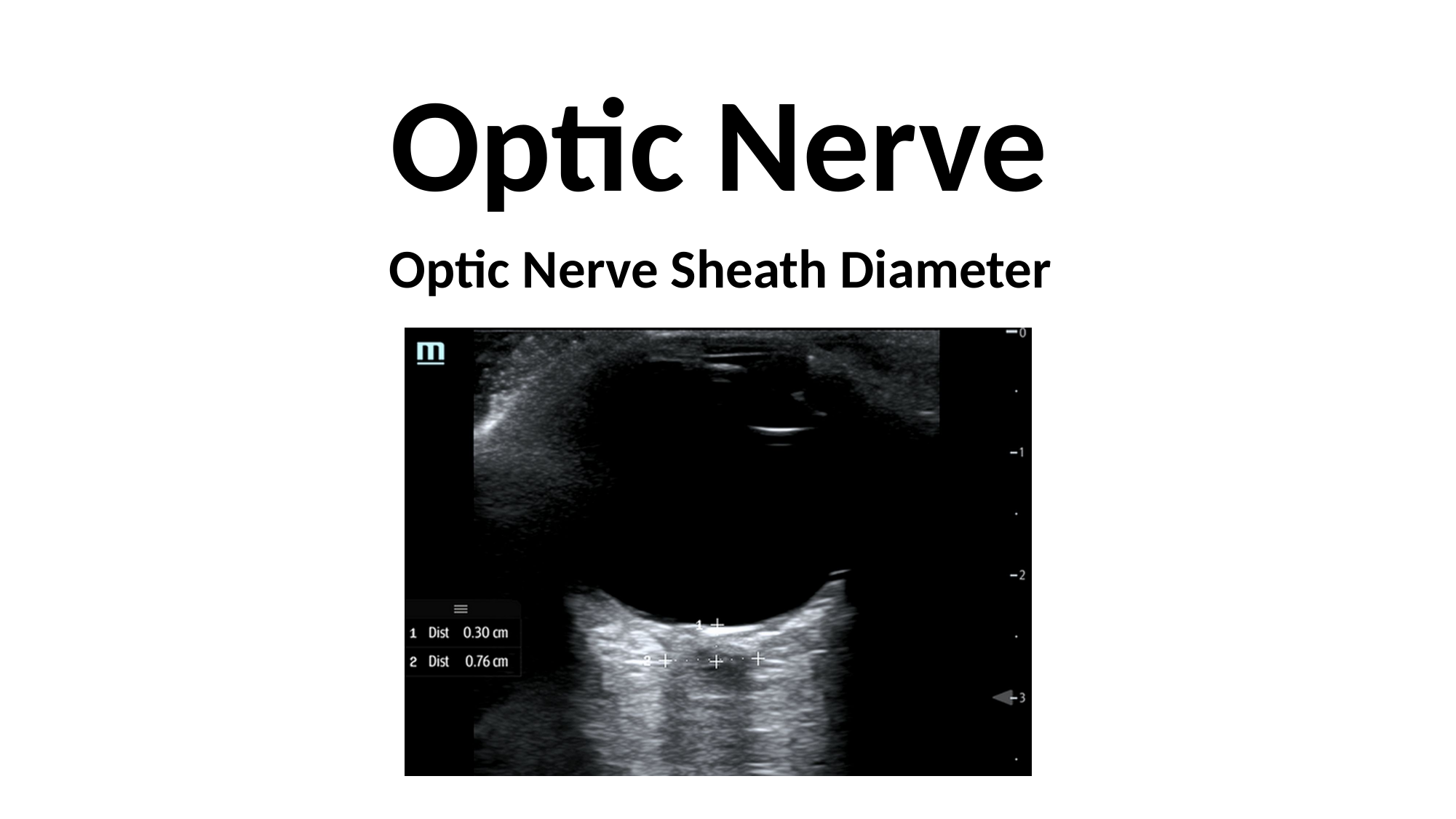

Optic Nerve
Optic Nerve Sheath Diameter

## Slide 24
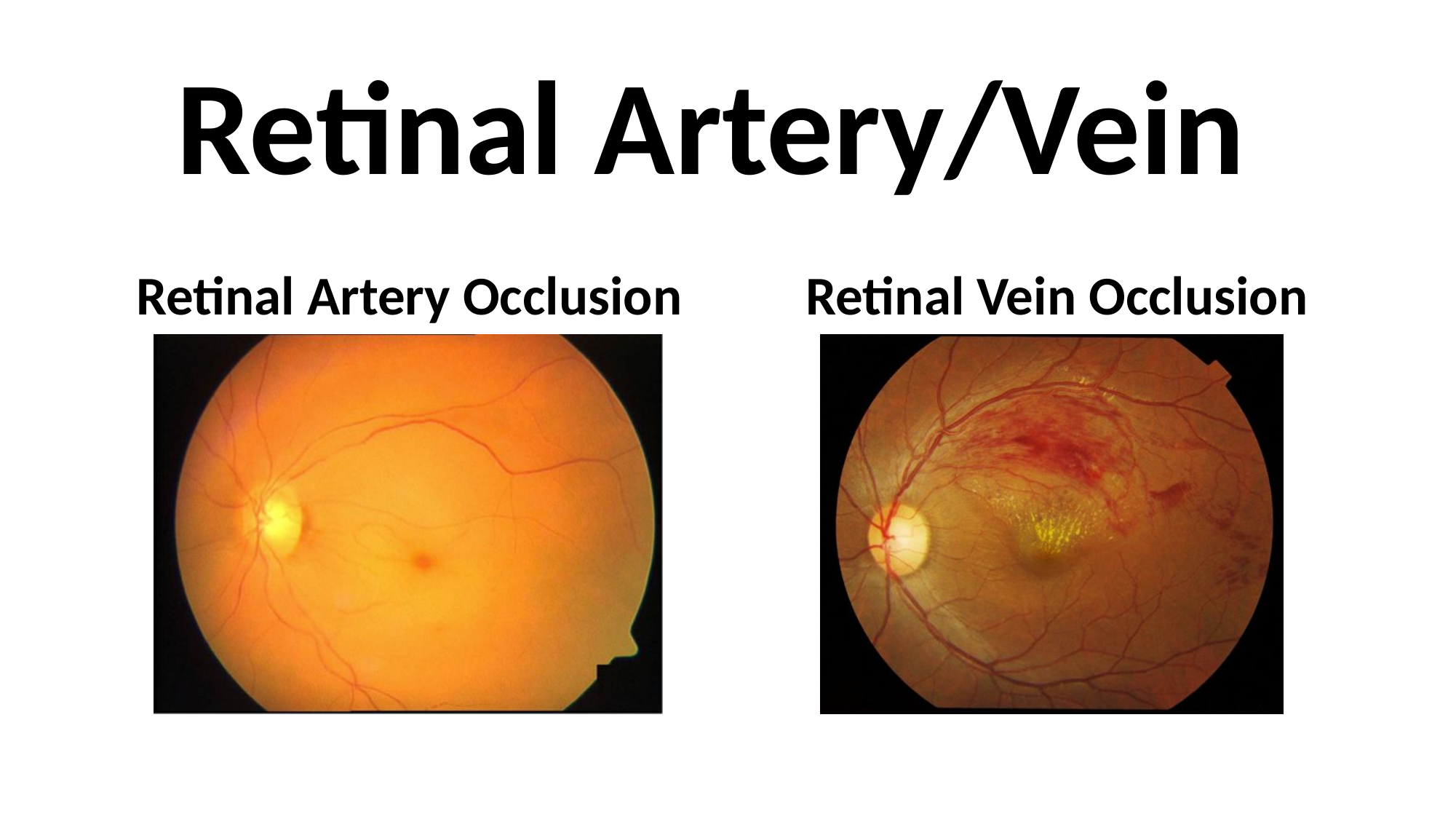

Retinal Artery/Vein
Retinal Artery Occlusion
Retinal Vein Occlusion

## Slide 25
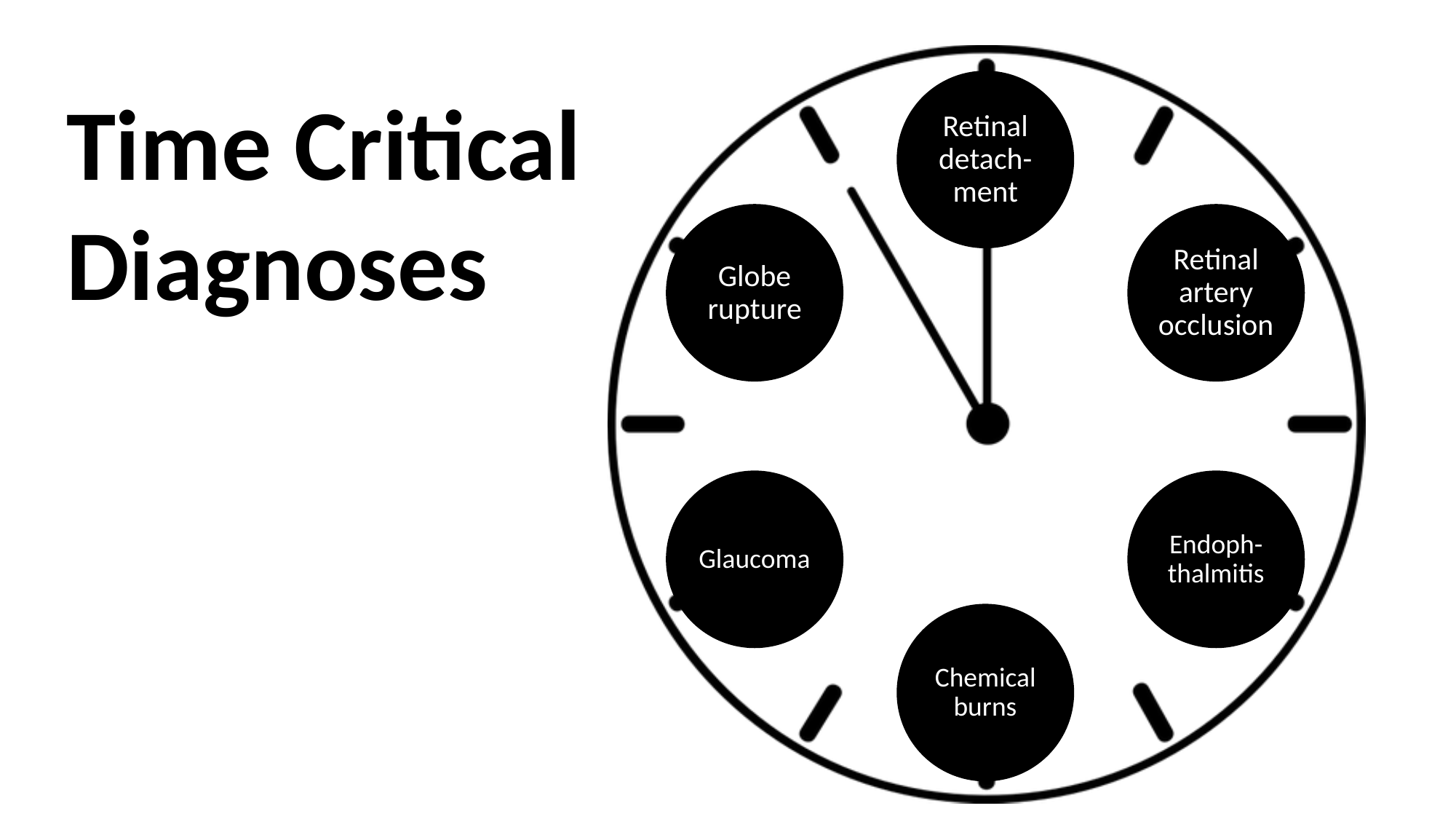

Time Critical Diagnoses

## Slide 26
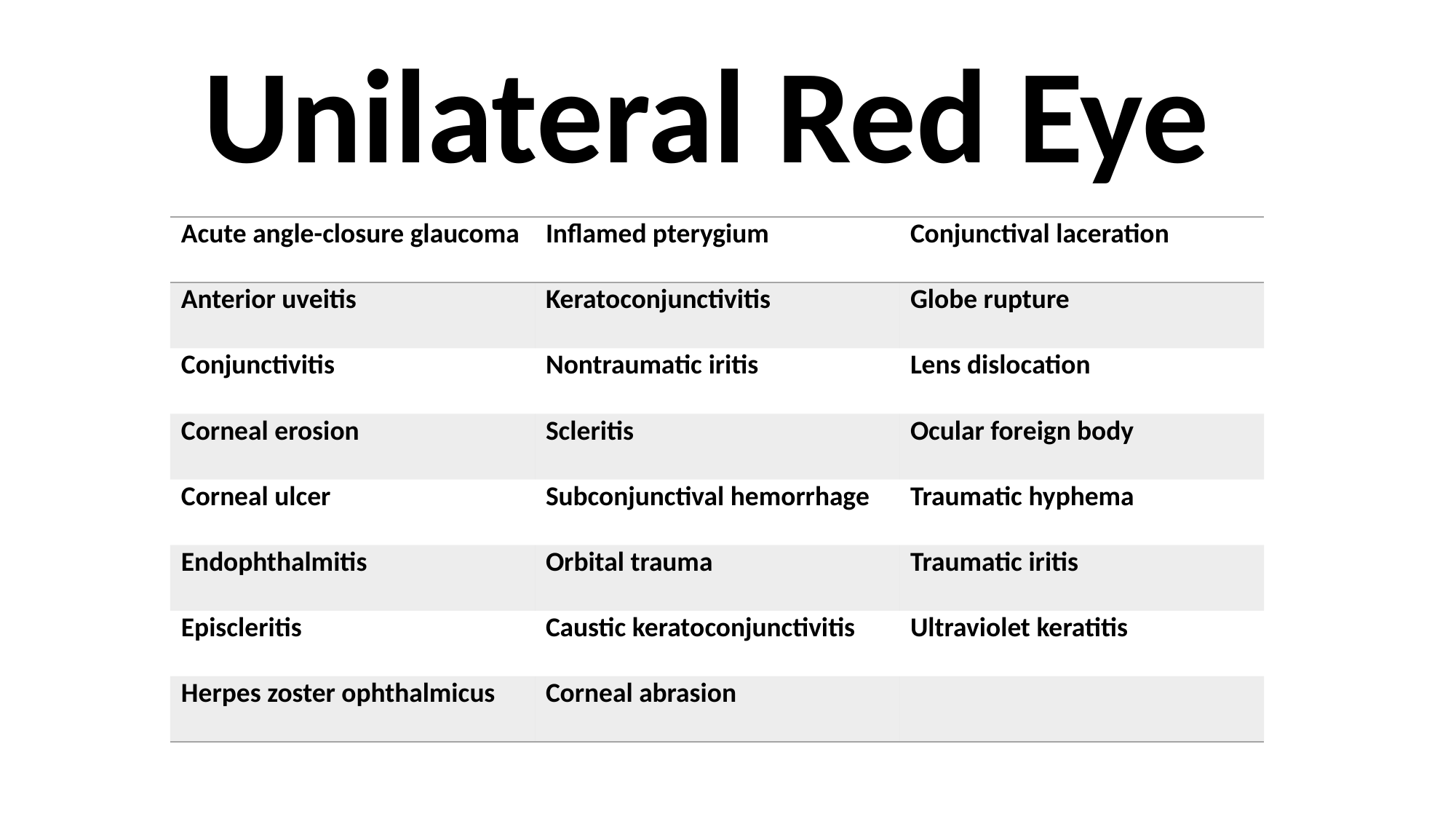

Unilateral Red Eye
| Acute angle-closure glaucoma | Inflamed pterygium | Conjunctival laceration |
| --- | --- | --- |
| Anterior uveitis | Keratoconjunctivitis | Globe rupture |
| Conjunctivitis | Nontraumatic iritis | Lens dislocation |
| Corneal erosion | Scleritis | Ocular foreign body |
| Corneal ulcer | Subconjunctival hemorrhage | Traumatic hyphema |
| Endophthalmitis | Orbital trauma | Traumatic iritis |
| Episcleritis | Caustic keratoconjunctivitis | Ultraviolet keratitis |
| Herpes zoster ophthalmicus | Corneal abrasion | |

## Slide 27
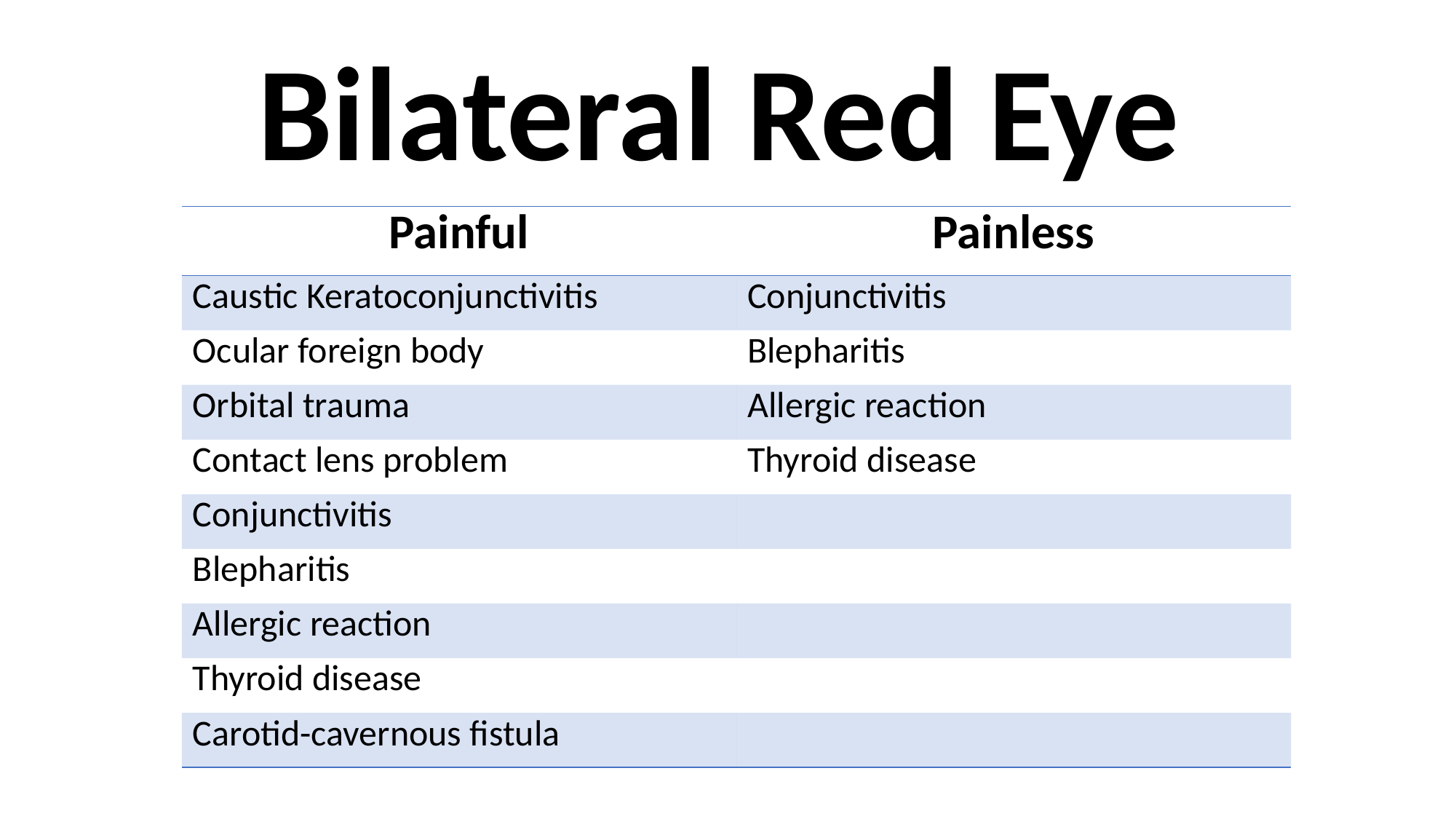

Bilateral Red Eye
| Painful | Painless |
| --- | --- |
| Caustic Keratoconjunctivitis | Conjunctivitis |
| Ocular foreign body | Blepharitis |
| Orbital trauma | Allergic reaction |
| Contact lens problem | Thyroid disease |
| Conjunctivitis | |
| Blepharitis | |
| Allergic reaction | |
| Thyroid disease | |
| Carotid-cavernous fistula | |

## Slide 28
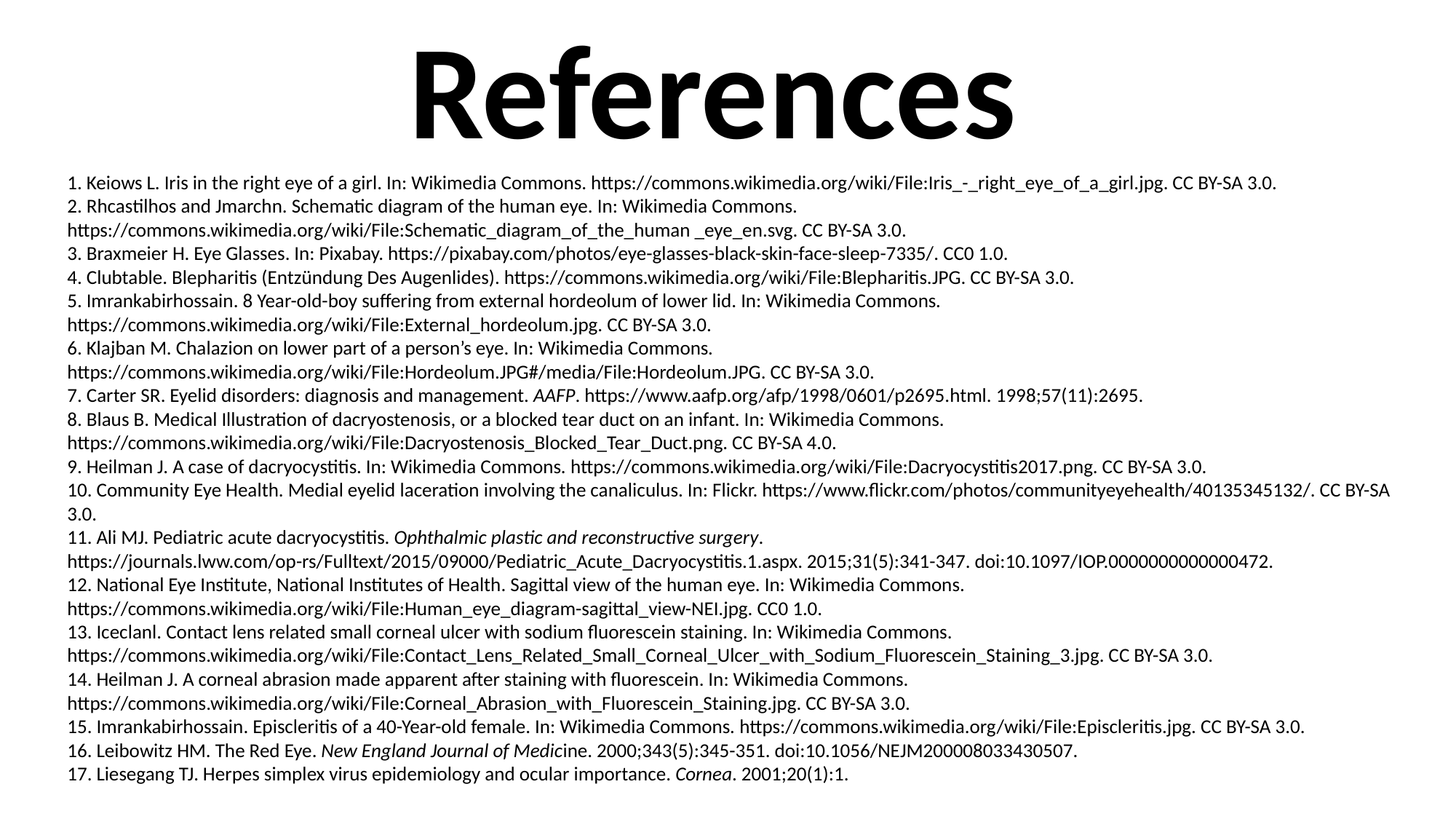

References
1. Keiows L. Iris in the right eye of a girl. In: Wikimedia Commons. https://commons.wikimedia.org/wiki/File:Iris_-_right_eye_of_a_girl.jpg. CC BY-SA 3.0.
2. Rhcastilhos and Jmarchn. Schematic diagram of the human eye. In: Wikimedia Commons. https://commons.wikimedia.org/wiki/File:Schematic_diagram_of_the_human _eye_en.svg. CC BY-SA 3.0.
3. Braxmeier H. Eye Glasses. In: Pixabay. https://pixabay.com/photos/eye-glasses-black-skin-face-sleep-7335/. CC0 1.0.
4. Clubtable. Blepharitis (Entzündung Des Augenlides). https://commons.wikimedia.org/wiki/File:Blepharitis.JPG. CC BY-SA 3.0.
5. Imrankabirhossain. 8 Year-old-boy suffering from external hordeolum of lower lid. In: Wikimedia Commons. https://commons.wikimedia.org/wiki/File:External_hordeolum.jpg. CC BY-SA 3.0.
6. Klajban M. Chalazion on lower part of a person’s eye. In: Wikimedia Commons. https://commons.wikimedia.org/wiki/File:Hordeolum.JPG#/media/File:Hordeolum.JPG. CC BY-SA 3.0.
7. Carter SR. Eyelid disorders: diagnosis and management. AAFP. https://www.aafp.org/afp/1998/0601/p2695.html. 1998;57(11):2695.
8. Blaus B. Medical Illustration of dacryostenosis, or a blocked tear duct on an infant. In: Wikimedia Commons. https://commons.wikimedia.org/wiki/File:Dacryostenosis_Blocked_Tear_Duct.png. CC BY-SA 4.0.
9. Heilman J. A case of dacryocystitis. In: Wikimedia Commons. https://commons.wikimedia.org/wiki/File:Dacryocystitis2017.png. CC BY-SA 3.0.
10. Community Eye Health. Medial eyelid laceration involving the canaliculus. In: Flickr. https://www.flickr.com/photos/communityeyehealth/40135345132/. CC BY-SA 3.0.
11. Ali MJ. Pediatric acute dacryocystitis. Ophthalmic plastic and reconstructive surgery. https://journals.lww.com/op-rs/Fulltext/2015/09000/Pediatric_Acute_Dacryocystitis.1.aspx. 2015;31(5):341-347. doi:10.1097/IOP.0000000000000472.
12. National Eye Institute, National Institutes of Health. Sagittal view of the human eye. In: Wikimedia Commons. https://commons.wikimedia.org/wiki/File:Human_eye_diagram-sagittal_view-NEI.jpg. CC0 1.0.
13. Iceclanl. Contact lens related small corneal ulcer with sodium fluorescein staining. In: Wikimedia Commons. https://commons.wikimedia.org/wiki/File:Contact_Lens_Related_Small_Corneal_Ulcer_with_Sodium_Fluorescein_Staining_3.jpg. CC BY-SA 3.0.
14. Heilman J. A corneal abrasion made apparent after staining with fluorescein. In: Wikimedia Commons. https://commons.wikimedia.org/wiki/File:Corneal_Abrasion_with_Fluorescein_Staining.jpg. CC BY-SA 3.0.
15. Imrankabirhossain. Episcleritis of a 40-Year-old female. In: Wikimedia Commons. https://commons.wikimedia.org/wiki/File:Episcleritis.jpg. CC BY-SA 3.0.
16. Leibowitz HM. The Red Eye. New England Journal of Medicine. 2000;343(5):345-351. doi:10.1056/NEJM200008033430507.
17. Liesegang TJ. Herpes simplex virus epidemiology and ocular importance. Cornea. 2001;20(1):1.

## Slide 29
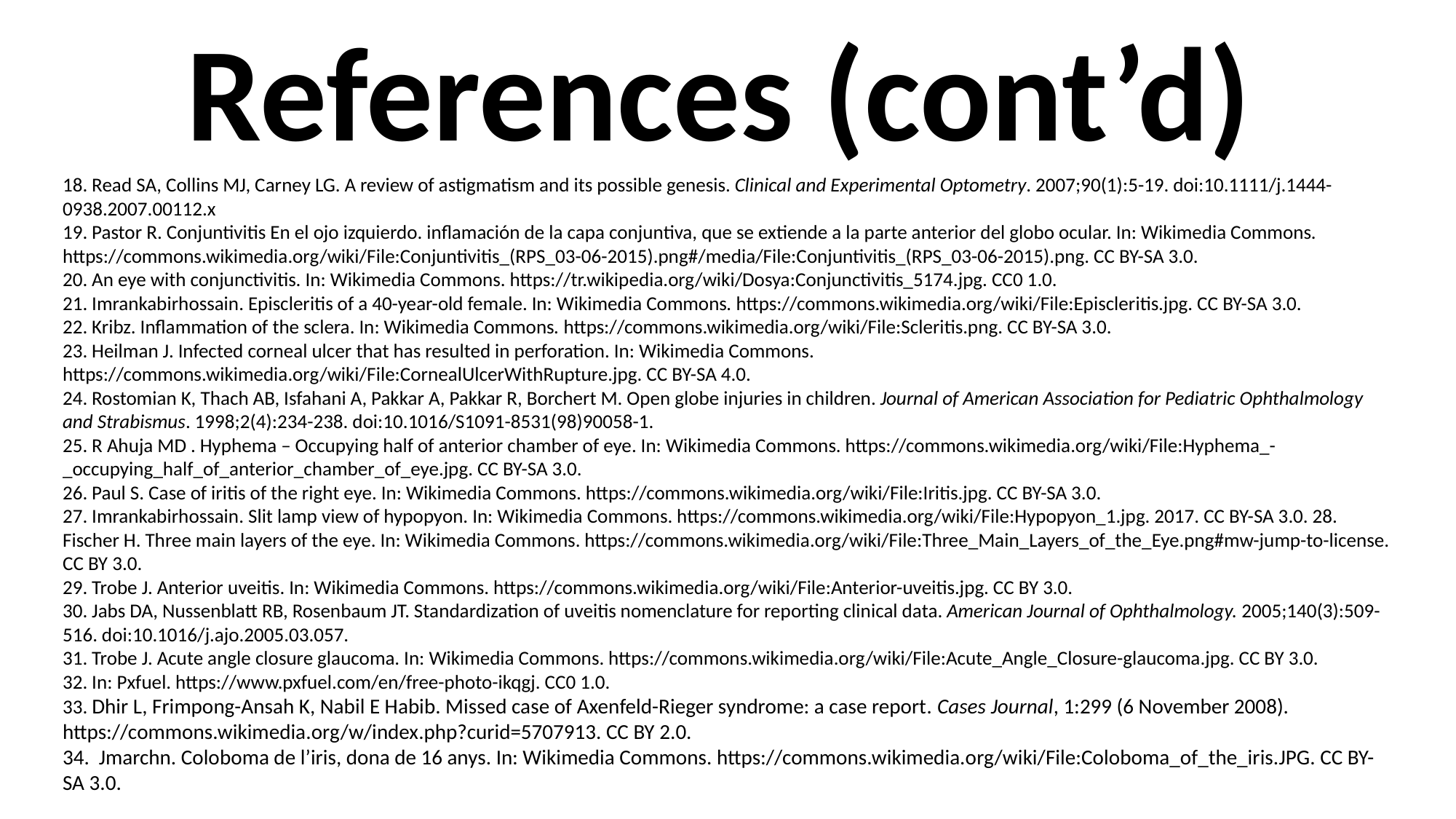

References (cont’d)
18. Read SA, Collins MJ, Carney LG. A review of astigmatism and its possible genesis. Clinical and Experimental Optometry. 2007;90(1):5-19. doi:10.1111/j.1444-0938.2007.00112.x
19. Pastor R. Conjuntivitis En el ojo izquierdo. inflamación de la capa conjuntiva, que se extiende a la parte anterior del globo ocular. In: Wikimedia Commons. https://commons.wikimedia.org/wiki/File:Conjuntivitis_(RPS_03-06-2015).png#/media/File:Conjuntivitis_(RPS_03-06-2015).png. CC BY-SA 3.0.
20. An eye with conjunctivitis. In: Wikimedia Commons. https://tr.wikipedia.org/wiki/Dosya:Conjunctivitis_5174.jpg. CC0 1.0.
21. Imrankabirhossain. Episcleritis of a 40-year-old female. In: Wikimedia Commons. https://commons.wikimedia.org/wiki/File:Episcleritis.jpg. CC BY-SA 3.0.
22. Kribz. Inflammation of the sclera. In: Wikimedia Commons. https://commons.wikimedia.org/wiki/File:Scleritis.png. CC BY-SA 3.0.
23. Heilman J. Infected corneal ulcer that has resulted in perforation. In: Wikimedia Commons. https://commons.wikimedia.org/wiki/File:CornealUlcerWithRupture.jpg. CC BY-SA 4.0.
24. Rostomian K, Thach AB, Isfahani A, Pakkar A, Pakkar R, Borchert M. Open globe injuries in children. Journal of American Association for Pediatric Ophthalmology and Strabismus. 1998;2(4):234-238. doi:10.1016/S1091-8531(98)90058-1.
25. R Ahuja MD . Hyphema – Occupying half of anterior chamber of eye. In: Wikimedia Commons. https://commons.wikimedia.org/wiki/File:Hyphema_-_occupying_half_of_anterior_chamber_of_eye.jpg. CC BY-SA 3.0.
26. Paul S. Case of iritis of the right eye. In: Wikimedia Commons. https://commons.wikimedia.org/wiki/File:Iritis.jpg. CC BY-SA 3.0.
27. Imrankabirhossain. Slit lamp view of hypopyon. In: Wikimedia Commons. https://commons.wikimedia.org/wiki/File:Hypopyon_1.jpg. 2017. CC BY-SA 3.0. 28. Fischer H. Three main layers of the eye. In: Wikimedia Commons. https://commons.wikimedia.org/wiki/File:Three_Main_Layers_of_the_Eye.png#mw-jump-to-license. CC BY 3.0.
29. Trobe J. Anterior uveitis. In: Wikimedia Commons. https://commons.wikimedia.org/wiki/File:Anterior-uveitis.jpg. CC BY 3.0.
30. Jabs DA, Nussenblatt RB, Rosenbaum JT. Standardization of uveitis nomenclature for reporting clinical data. American Journal of Ophthalmology. 2005;140(3):509-516. doi:10.1016/j.ajo.2005.03.057.
31. Trobe J. Acute angle closure glaucoma. In: Wikimedia Commons. https://commons.wikimedia.org/wiki/File:Acute_Angle_Closure-glaucoma.jpg. CC BY 3.0.
32. In: Pxfuel. https://www.pxfuel.com/en/free-photo-ikqgj. CC0 1.0.
33. Dhir L, Frimpong-Ansah K, Nabil E Habib. Missed case of Axenfeld-Rieger syndrome: a case report. Cases Journal, 1:299 (6 November 2008). https://commons.wikimedia.org/w/index.php?curid=5707913. CC BY 2.0.
34. Jmarchn. Coloboma de l’iris, dona de 16 anys. In: Wikimedia Commons. https://commons.wikimedia.org/wiki/File:Coloboma_of_the_iris.JPG. CC BY-SA 3.0.

## Slide 30
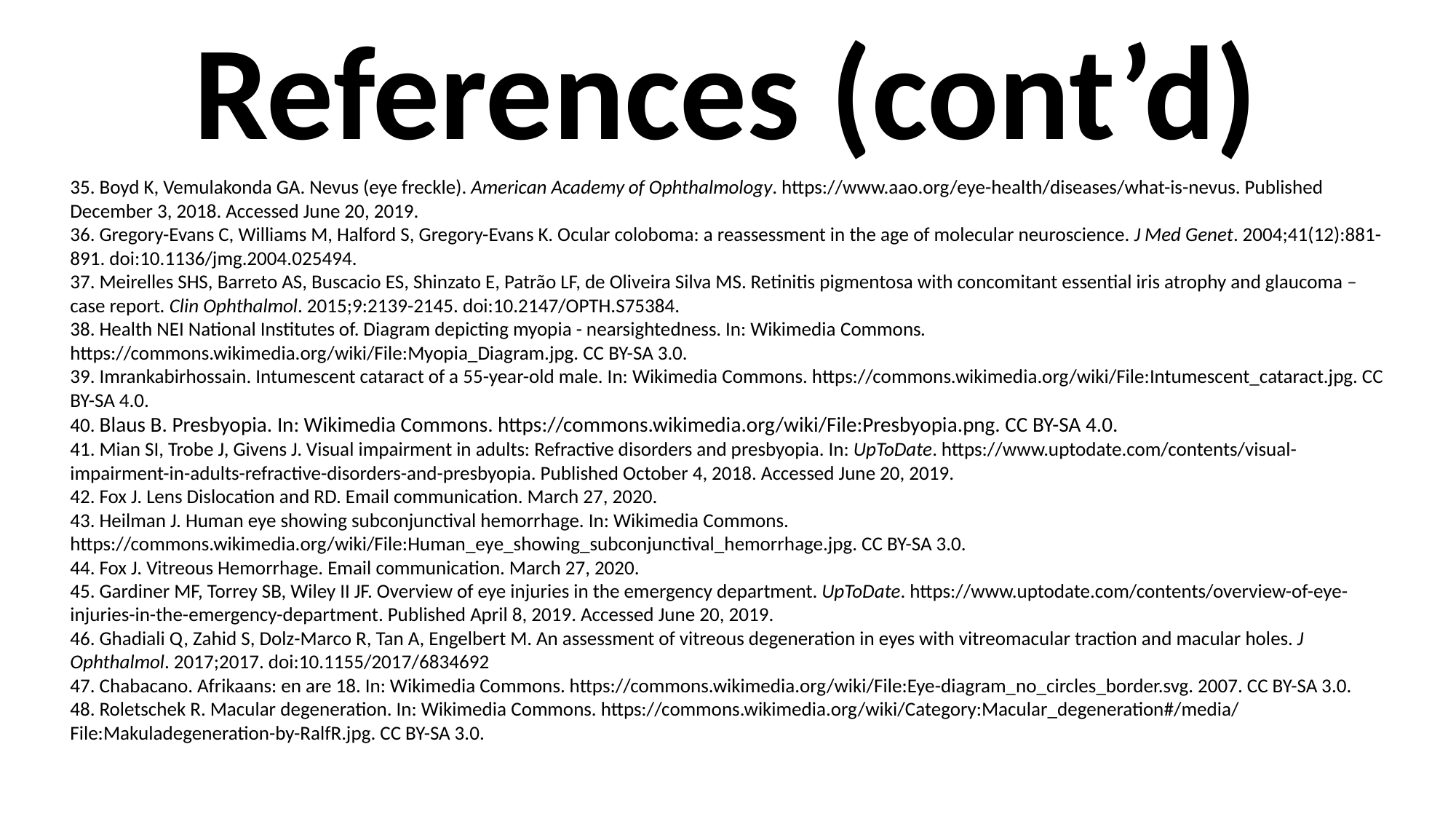

References (cont’d)
35. Boyd K, Vemulakonda GA. Nevus (eye freckle). American Academy of Ophthalmology. https://www.aao.org/eye-health/diseases/what-is-nevus. Published December 3, 2018. Accessed June 20, 2019.
36. Gregory-Evans C, Williams M, Halford S, Gregory-Evans K. Ocular coloboma: a reassessment in the age of molecular neuroscience. J Med Genet. 2004;41(12):881-891. doi:10.1136/jmg.2004.025494.
37. Meirelles SHS, Barreto AS, Buscacio ES, Shinzato E, Patrão LF, de Oliveira Silva MS. Retinitis pigmentosa with concomitant essential iris atrophy and glaucoma – case report. Clin Ophthalmol. 2015;9:2139-2145. doi:10.2147/OPTH.S75384.
38. Health NEI National Institutes of. Diagram depicting myopia - nearsightedness. In: Wikimedia Commons. https://commons.wikimedia.org/wiki/File:Myopia_Diagram.jpg. CC BY-SA 3.0.
39. Imrankabirhossain. Intumescent cataract of a 55-year-old male. In: Wikimedia Commons. https://commons.wikimedia.org/wiki/File:Intumescent_cataract.jpg. CC BY-SA 4.0.
40. Blaus B. Presbyopia. In: Wikimedia Commons. https://commons.wikimedia.org/wiki/File:Presbyopia.png. CC BY-SA 4.0.
41. Mian SI, Trobe J, Givens J. Visual impairment in adults: Refractive disorders and presbyopia. In: UpToDate. https://www.uptodate.com/contents/visual-impairment-in-adults-refractive-disorders-and-presbyopia. Published October 4, 2018. Accessed June 20, 2019.
42. Fox J. Lens Dislocation and RD. Email communication. March 27, 2020.
43. Heilman J. Human eye showing subconjunctival hemorrhage. In: Wikimedia Commons. https://commons.wikimedia.org/wiki/File:Human_eye_showing_subconjunctival_hemorrhage.jpg. CC BY-SA 3.0.
44. Fox J. Vitreous Hemorrhage. Email communication. March 27, 2020.
45. Gardiner MF, Torrey SB, Wiley II JF. Overview of eye injuries in the emergency department. UpToDate. https://www.uptodate.com/contents/overview-of-eye-injuries-in-the-emergency-department. Published April 8, 2019. Accessed June 20, 2019.
46. Ghadiali Q, Zahid S, Dolz-Marco R, Tan A, Engelbert M. An assessment of vitreous degeneration in eyes with vitreomacular traction and macular holes. J Ophthalmol. 2017;2017. doi:10.1155/2017/6834692
47. Chabacano. Afrikaans: en are 18. In: Wikimedia Commons. https://commons.wikimedia.org/wiki/File:Eye-diagram_no_circles_border.svg. 2007. CC BY-SA 3.0.
48. Roletschek R. Macular degeneration. In: Wikimedia Commons. https://commons.wikimedia.org/wiki/Category:Macular_degeneration#/media/File:Makuladegeneration-by-RalfR.jpg. CC BY-SA 3.0.

## Slide 31
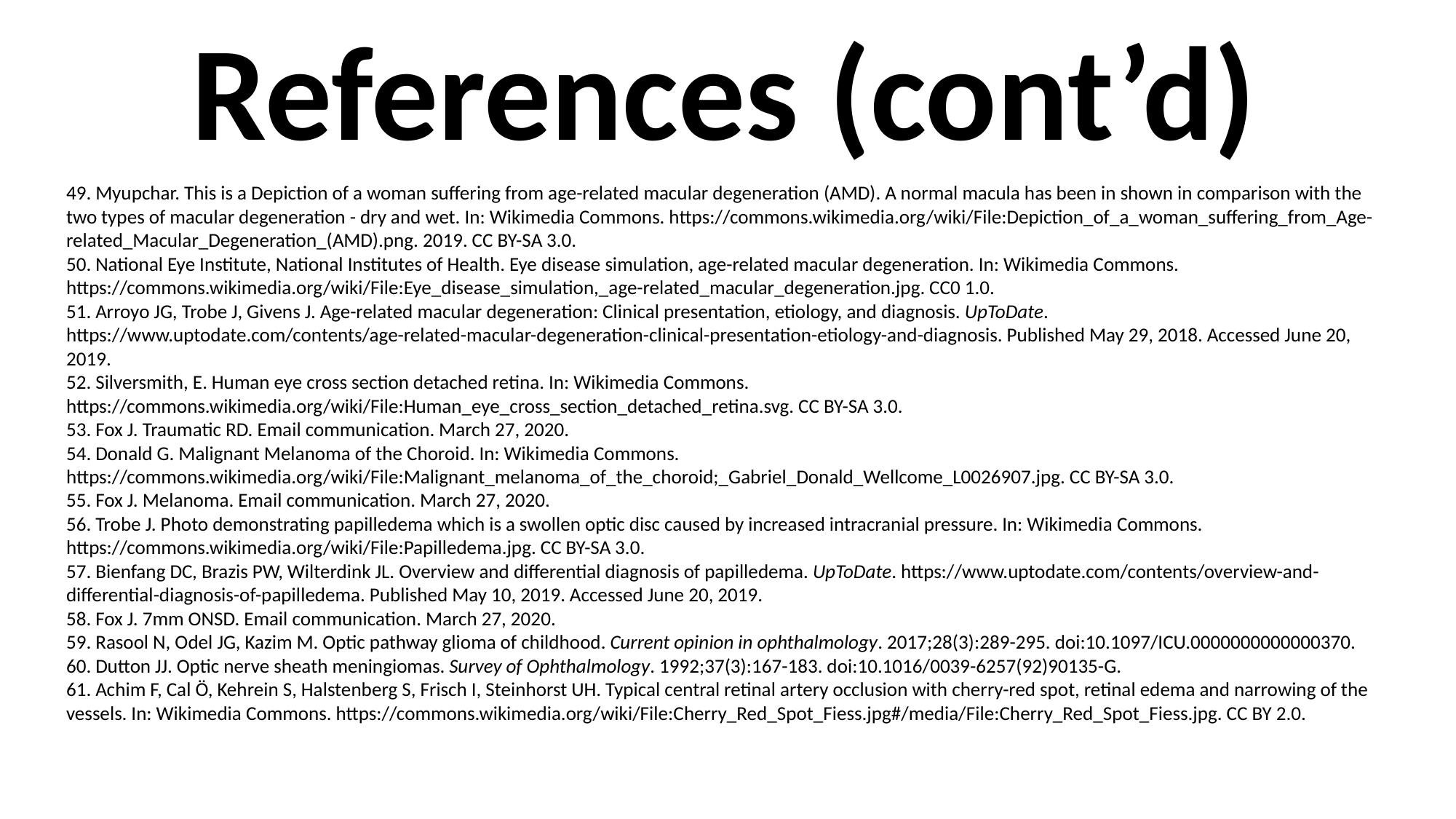

References (cont’d)
49. Myupchar. This is a Depiction of a woman suffering from age-related macular degeneration (AMD). A normal macula has been in shown in comparison with the two types of macular degeneration - dry and wet. In: Wikimedia Commons. https://commons.wikimedia.org/wiki/File:Depiction_of_a_woman_suffering_from_Age-related_Macular_Degeneration_(AMD).png. 2019. CC BY-SA 3.0.
50. National Eye Institute, National Institutes of Health. Eye disease simulation, age-related macular degeneration. In: Wikimedia Commons. https://commons.wikimedia.org/wiki/File:Eye_disease_simulation,_age-related_macular_degeneration.jpg. CC0 1.0.
51. Arroyo JG, Trobe J, Givens J. Age-related macular degeneration: Clinical presentation, etiology, and diagnosis. UpToDate. https://www.uptodate.com/contents/age-related-macular-degeneration-clinical-presentation-etiology-and-diagnosis. Published May 29, 2018. Accessed June 20, 2019.
52. Silversmith, E. Human eye cross section detached retina. In: Wikimedia Commons. https://commons.wikimedia.org/wiki/File:Human_eye_cross_section_detached_retina.svg. CC BY-SA 3.0.
53. Fox J. Traumatic RD. Email communication. March 27, 2020.
54. Donald G. Malignant Melanoma of the Choroid. In: Wikimedia Commons. https://commons.wikimedia.org/wiki/File:Malignant_melanoma_of_the_choroid;_Gabriel_Donald_Wellcome_L0026907.jpg. CC BY-SA 3.0.
55. Fox J. Melanoma. Email communication. March 27, 2020.
56. Trobe J. Photo demonstrating papilledema which is a swollen optic disc caused by increased intracranial pressure. In: Wikimedia Commons. https://commons.wikimedia.org/wiki/File:Papilledema.jpg. CC BY-SA 3.0.
57. Bienfang DC, Brazis PW, Wilterdink JL. Overview and differential diagnosis of papilledema. UpToDate. https://www.uptodate.com/contents/overview-and-differential-diagnosis-of-papilledema. Published May 10, 2019. Accessed June 20, 2019.
58. Fox J. 7mm ONSD. Email communication. March 27, 2020.
59. Rasool N, Odel JG, Kazim M. Optic pathway glioma of childhood. Current opinion in ophthalmology. 2017;28(3):289-295. doi:10.1097/ICU.0000000000000370.
60. Dutton JJ. Optic nerve sheath meningiomas. Survey of Ophthalmology. 1992;37(3):167-183. doi:10.1016/0039-6257(92)90135-G.
61. Achim F, Cal Ö, Kehrein S, Halstenberg S, Frisch I, Steinhorst UH. Typical central retinal artery occlusion with cherry-red spot, retinal edema and narrowing of the vessels. In: Wikimedia Commons. https://commons.wikimedia.org/wiki/File:Cherry_Red_Spot_Fiess.jpg#/media/File:Cherry_Red_Spot_Fiess.jpg. CC BY 2.0.

## Slide 32
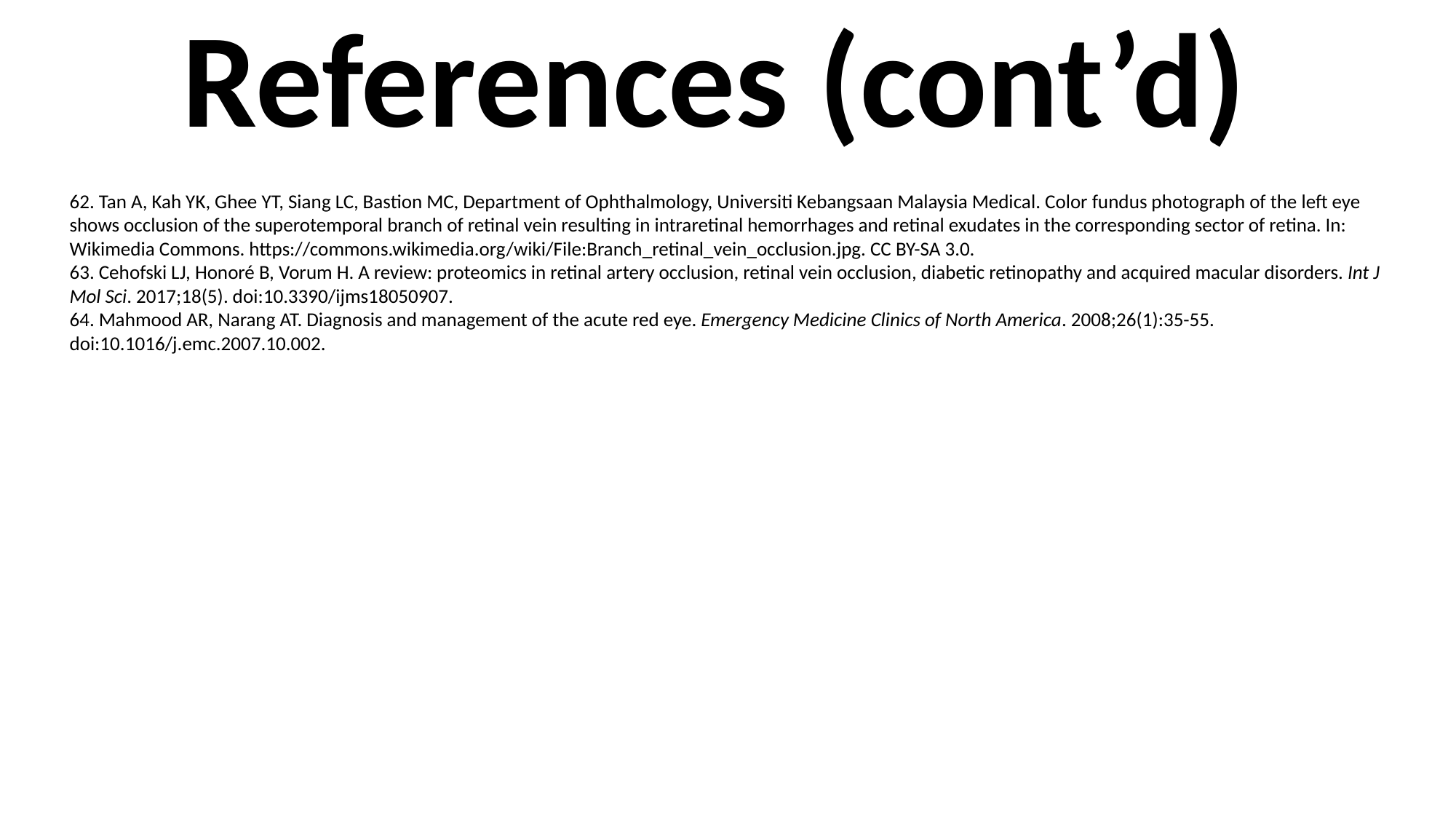

# References (cont’d)
62. Tan A, Kah YK, Ghee YT, Siang LC, Bastion MC, Department of Ophthalmology, Universiti Kebangsaan Malaysia Medical. Color fundus photograph of the left eye shows occlusion of the superotemporal branch of retinal vein resulting in intraretinal hemorrhages and retinal exudates in the corresponding sector of retina. In: Wikimedia Commons. https://commons.wikimedia.org/wiki/File:Branch_retinal_vein_occlusion.jpg. CC BY-SA 3.0.
63. Cehofski LJ, Honoré B, Vorum H. A review: proteomics in retinal artery occlusion, retinal vein occlusion, diabetic retinopathy and acquired macular disorders. Int J Mol Sci. 2017;18(5). doi:10.3390/ijms18050907.
64. Mahmood AR, Narang AT. Diagnosis and management of the acute red eye. Emergency Medicine Clinics of North America. 2008;26(1):35-55. doi:10.1016/j.emc.2007.10.002.
